# The Effect of Microwave Oven Extraction Temperature, Time, and Power Optimization on the Determination of Heavy Metals in Apricot by ICP-MS

**DOI:** 10.1021/acsomega.5c13020

**Published:** 2026-06-18

**Authors:** Servet Askin, Nevin Çankaya, Halim Yılmaz

**Affiliations:** † Vocational School of Health Services, 187440 Igdir University, IĞdır 76100, Turkey; ‡ Vocational School of Health Services, Usak University, Usak 64200, Turkey; § Postgraduate Education Institute, Igdir University, IĞdır 76100, Turkey

## Abstract

This paper investigates the concentrations of heavy metals in apricots, which are widely consumed as food and may pose serious health risks when exceeding permissible limits. The elements analyzed included lead (Pb), copper (Cu), zinc (Zn), arsenic (As), selenium (Se), cadmium (Cd), and mercury (Hg). The effects of microwave optimization digestion (MWOD) parameters, including temperature (tp), time (tm), and power (pw), on the extraction efficiency of these elements were systematically evaluated in apricot kernel (K), kernel shell (S), and flesh (F) samples. Microwave digestion was performed according to EPA Methods 3051A and 3052. Following digestion and complete solubilization, the concentrations of heavy metals were determined using inductively coupled plasma mass spectrometry (ICP-MS). The highest concentrations of Cd and Hg in apricot flesh were found to be 25.49 μg kg^–1^ and 14.21 μg kg^–1^ under optimized digestion conditions of 1000 W at 220 °C for 20 min and 800 W at 220 °C for 20 min, respectively. Pearson correlation analysis revealed a strong positive relationship between Zn and Cu under the applied digestion conditions. The accuracy and reliability of the analytical results were validated using the certified reference material BCR-414 (plankton). In addition, the potential health risks associated with the detected heavy metals were assessed using Estimated Daily Intake (EDI) and Hazard Quotient (HQ) models. The optimization of microwave digestion parameters significantly influenced extraction efficiency, and the combined use of optimized digestion and ICP-MS analysis enabled reliable identification and quantification of toxic elements in different apricot partitions.

## Graphical abstract

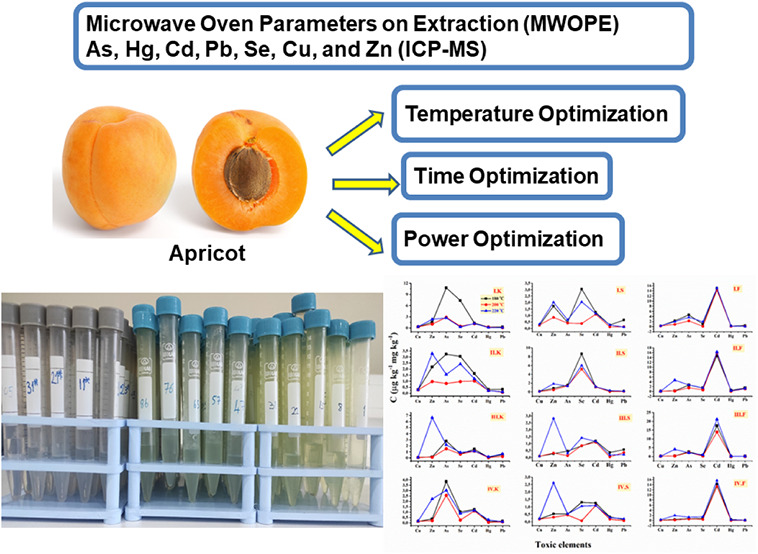

## 1 Introduction

Soil, water, and air contain essential elements vital for various biological processes, as well as nonessential metals such as cadmium (Cd) and lead (Pb), which can be toxic even in relatively low concentrations.^1–3^ These are pollutants that are not biodegradable, can accumulate in soil environments, and can migrate.^3^

Foods are complex matrices composed of essential nutrients, non-nutritive components, and potentially toxic substances, including metals.^4^ Heavy metals such as mercury, lead, chromium, cadmium, and arsenic exhibit well-documented toxic mechanisms.^5^ Among the many different minerals that we consume in our daily diet, particular attention should be paid to toxic heavy metals such as Cd, Pb, Hg, or Ni. These elements have no known positive functions and can have toxic effects even in relatively low concentrations.^5,6^ Food metal contamination is a global public health problem because dietary metals represent one of the primary ways people are exposed to these contaminants.^7–9^

Apricot (*Prunus armeniaca* L.) is a species belonging to the genus *Prunus*, the family Rosaceae, and the order Rosales.^10^ The apricot is a drupe in structure. Anatomically, it consists of four parts: the epicarp, mesocarp, endocarp, and kernel.^10–13^ Heavy metals such as As, Hg, Cd, Se, Zn, Pb, and Cu in apricots, which are consumed as fruit and nuts, can accumulate in the human body during consumption and lead to central nervous system disorders, skeletal system disabilities (osteodystrophy), urinary symptoms associated with renal failure, liver diseases and cancer, gastrointestinal disorders, hypertension, mental disorders in children in early periods, and ulceration.^14–19^ As a result of the MWOD process of foods, Zor found heavy metals in spinach, lettuce, and parsley sold in the Marmara region.^20^ In their study, TokalıoĞlu et al., found heavy metals in flaked red pepper^21^ and Pardinho et al., identified trace elements in the Yerba mate plant by ICP-MS.^22^ Umaz et al. found heavy metals in the topsoil parts of some plant species,^23^ and Magalhâesa et al. identified heavy metals in edible, chia, flax, and coconut oils by ICP-MS measurement as a result of the MWOD process.^24^ A study conducted by Chatterjee reported arsenic compounds in oyster tissue.^25^ Ashoka et al. found trace elements in fish tissue samples,^26^ Lao et al. identified heavy metals in seafood,^27^ and Türkmen et al. found trace elements in fish species in the Mediterranean and Aegean seas by ICP-MS as a result of MWOD.^28^ In studies conducted by Öztürk and Lee et al., using the MWOD process with cow’s milk, they determined major and minor elements in cow’s milk by ICP-MS.^29,30^ These studies demonstrate the effectiveness of MWOD coupled with ICP-MS for trace element analysis across diverse food matrices. Using the MWOD process, Lee et al. found minor and major elements in rice in Brazil,^31^ and Barnet et al. identified minor and major elements in rice in South Korea by ICP-MS.^32^ Shishov et al. found major and minor elements in tobacco and lettuce leaves,^33^ Bhandari and Amarasiriwardena found trace elements in maple sap,^34^ and Sucharova and Suchara found major and minor elements in peach leaves, spinach, pine leaves, aquatic plants, and lagarosiphon by ICP-MS as a result of the MWOD process.^35^ Using the MWOD process, TokalıoĞlu found major and minor elements in medicinal herbs,^36^ Barbosa et al. detected trace elements in soybean products,^37^ Komorowicz et al. identified arsenic in mushroom species,^38^ and Bengü et al. found major and minor elements in honey by ICP-MS.^39^

In microwave-assisted ultraviolet (UV) acid digestion, Hartwig et al. identified Ni, Pd, and Pt elements as catalysts in the production of margarine oils,^40^ and Aydin identified major and minor elements in wool by ICP-MS-OES.^41^ In a study conducted by Chen et al. on the quantitative identification of major and minor elements in oranges, they used the MWOD process and the ICP-MS device.^42^

This study analyzed major and minor elements separately in orange seeds, orange pulp, and orange peel. The results showed that orange is rich in Mg, K, and Ca elements. The highest amount of Mg was found in orange peel, while the highest amount of K element was found in the seeds. As, Cd, and Pb elements were found at low concentrations in orange. The wet digestion method was used for the identification of K, Mg, Ca, Cu, Zn, Fe, and Mn minerals in IĞdır apricot flesh by AAS, and the following concentrations were found, respectively: K: 262.20–144.86, Mg: 14.54–8.39, Ca: 13.66–7.53, Cu: 0.27–0.11, Zn: 0.15–0.06, Fe: 1.06–0.37, and Mn: 0.09–0.04 mg 100 g^–1^; whereas they were 74.5–467.6, 164.53–126.86, 40.63–30.46, 1.51–1.05, 2.39–2.01, 2.91–2.11, 0.53–0.36 mg 100g^–1^, respectively, in the apricot kernel.^43^

The statistical analyses of Fe, Al, Mn, Zn, Cu, Pb, As, and Cd, which are metals present in the composition of red sweet pepper, showed significance at the 95% or 99% confidence level among the metal elements.^21^ In another study conducted on the determination of Cr, Mn, Fe, Ni, Co, As, Cd, Pb, and Zn elements in different spices, a significance level of 95–99% confidence interval was observed, and a correlation was detected.^44^ In the analysis of trace elements in rice consumed in South Korea, it was determined that brown rice contained higher levels of Al, As, Ba, Ca, Co, Fe, K, Mg, Mn, Ni, P, S, Se, and Zn compared to white rice (*p* < 0.001, *p* < 0.05), while Cd, Cr, Cu, and Pb (*p* > 0.05) showed no significant difference .^31^ In addition, one-way analysis of variance was used to analyze major and minor elements in milk (Al, As, Ba, Ca, Cd, Co, Cr, Cu, Fe, Mg, Mn, Ni, Pb, Sb, Se, and Zn).^29,30^ On the other hand, in the investigation of heavy metal elements in raw milk, Cu, Fe, Zn, Cr, Ni, Cd, As, and Pb were determined using Duncan’s multiple range test (*p* < 0.05).

In the determination of Pb, Cd, Hg, and As in five different fish species, the *p*-value was determined to be between 0.05 and 0.01 using the Student’s *t*-test for independent samples by analysis of variance.^45^ In the study investigating the physical, chemical, and mechanical properties of some apricot varieties cultivated in Türkiye, no statistical evaluation was carried out to determine the minerals in the apricot composition.^10–12^

Although there has been no research in the literature on the toxic properties of Pb, Cu, Zn, As, Se, Cd, and Hg elements found in apricot seeds, seed shells, and fruit pulp in organisms, studies have been conducted on the toxicity of heavy metals in fish and mushrooms.^46–48^ In a study on the detection of toxic elements Pb, As, Cd, and Hg in the composition of cereals consumed, it is explained that they pose a risk to human health in the organism (metabolism).^49–51^ Furthermore, considering the toxic elements in foods and the limits that cause toxicity according to the World Health Organization, the epidemiological effect on public health is closely related to metabolic processes. Although there is no study on the toxic elements in apricots related to human health, the epidemiological effect of phenolic substances on health, especially in apricot seeds, has been investigated in many studies.^19,52–55^ The fact that this apricot (şalak) variety has been registered as a geographically indicated fruit by the Turkish Patent Institute and that the region where it is grown has volcanic rock characteristics adds a special value to this study.^56,68^

In this context, the present study applies EPA Methods 3051A^57^ and 3052 ^58^ to determine heavy metal concentrations in the Şalak apricot variety. Microwave digestion parameters (time, temperature, and power) were optimized, and Pb, Cu, Zn, As, Se, Cd, and Hg concentrations were quantified using ICP-MS. Statistical analyses were performed using SPSS to evaluate differences among apricot parts. Furthermore, the study aims to assess potential synergistic or additive effects of heavy metals and to evaluate associated health risks using Estimated Daily Intake (EDI) and Hazard Quotient (HQ) models.^54,55,59,60^

## 2 Material and Method

### 2.1 Study Area

The province of IĞdır, where the Şalak apricot species investigated in this study is cultivated, has an average altitude of 850 m, a plain area of 832 km^2^, and a microclimatic structure. The samples were collected from four locations: Aralık, Karakoyunlu, central IĞdır, and Tuzluca during the harvest season.

### 2.2 Sample Collection and Pre-Treatments

A total of ten ripe Şalak samples were randomly collected from four different trees in each study area to achieve homogeneity. They were then washed in ultradistilled water and grouped as apricot kernel, kernel shell, and flesh. Samples collected from four sites were dried in an oven at 70 °C for 72 h. The kernel and flesh parts of the dried apricot samples were ground in a mortar, and the kernel shells were ground with a jaw crusher in the Civil Engineering Laboratory at IĞdır University. The ground samples were further dried at 70 °C for 24 h, brought to constant weight in a desiccator, and then stored for analysis.

### 2.3 Chemicals and Tools Used

Apricot samples were digested using a microwave digestion system. Suprapure reagents including 65% HNO_3_, 36.5% HCl, and 30% H_2_O_2_ (Merck, Darmstadt, Germany) were used. The microwave system (ETHOS One, Milestone, Italy) consisted of polytetrafluoroethylene (PTFE)-sealed vessels (10–100 mL). Optimum extraction was achieved by microwave digestion (MWOD) with concentrated acid, using temperature (tp), time (tm), and power (pw) parameters in sealed containers. All the analyses were performed by the multielement method using an Agilent 7700 model (Agilent Corporation, USA) and inductively coupled plasma–mass spectrometry (ICP-MS). Measurement parameters obtained using ICP-MS are given in Table 1, while microwave digestion parameters are given in Tables 5, 8, and 11.

**Table 1:** Measurement Parameters of Apricot Extracts Measured Using the Agilent 7700 ICP-MS Model

Nebulizer	Babington Type
Spray chamber	Quarts, double pass
RF generator	Frequency: 10 MHz, power output: 1550 W
Ar flow rate (L/min)	0.96
Auxiliary gas flow rate (L/min)	0.88
nebulizer pressure (bar)	3.01
Number o f replicates	3
Integration time (s)	0.01
Internal standards	Sc, Ge, Lu, In, Bi
Isotopes	^63^Cu, ^66^Zn, ^75^As, ^78^Se, ^111^Cd, ^201^Hg, ^208^Pb

### 2.4 Reagents and Standards

The present study not only investigated variations in acid ratios used in EPA Methods 3051A and 3052 (U.S. Environmental Protection Agency) for the determination of heavy metals in apricot kernel, kernel shell, and flesh, but also examined the effects of microwave digestion parameters on extraction efficiency.^57,58^

We used As, Pb, Cd, Se, Cu, and Zn: 10 μg mL^–1^ in 5% (v/v) HNO_3_ Inorganic Ventures (Christiansburg, VA, USA), brand IV-STOCK-6 multimix standard solution, and Hg: 10 μg L^–1^ in 10% (v/v) HNO_3_ Inorganic Ventures brand MSHGN-10 ppm of Hg standard solution (Christiansburg, VA, USA) as primary standards. ISTD was added to the samples online. Inorganic Ventures brand IV-STOCK-75 coded standard solution was used with a 100× dilution as an ISTD standard. All dilutions were prepared using 10% HNO_3_ (aq) and 0.5% HCl (aq) with ASTM Type 1 ultrapure water. Table 2 presents the calibration concentrations prepared for each element for ICP-MS analysis (Supporting Information 5).

**Table 2:** Calibration Parameter Values for ICP-MS Measurement

Elem/const.										
**As** (μg kg^–1^)	0	5.000	10.000	25.000	50 × 10^3^	100 × 10^3^	500 × 10^3^	1 × 10^6^	5 × 10^6^	10 × 10^6^
RSD	16	2.1	3.6	2	8.7	2.4	0.9	0.2	1.5	0.2
**Hg** (μg kg^–1^)	0	0.5	1.000	2.500	5 × 10^3^	10 × 10^3^	-	-	-	-
RSD	7.5	3.9	2.3	2.9	1.4	2.5	0.8	9.5	14	6.2
**Cd** (μg kg^–1^)	0	0.5	1.000	2.500	5 × 10^3^	10 × 10^3^	50 × 10^3^	100 × 10^3^	500 × 10^3^	1 × 10^6^
RSD	5	2	3	2	1	2	1	2	6	2
**Pb** (μg kg^–1^)	.	-	-	2.500	5 × 10^3^	10 × 10^3^	50 × 10^3^	100 × 10^3^	500 × 10^3^	1 × 10^6^
RSD	4	2	1	1	0	3	2	1	6	0
**Se** (μg kg^–1^)	0	5.000	10.000	25.000	50 × 10^3^	100 × 10^3^	500 × 10^3^	1 × 10^6^	5 × 10^6^	10 × 10^6^
RSD	40	8.5	12	2.7	4.6	3.9	0.8	0.5	1.2	0.4
**Cu** (μg kg^–1^)	0	0.5	1.000	2.500	5 × 10^3^	10 × 10^3^	50 × 10^3^	100 × 10^3^	500 × 10^3^	1 × 10^6^
RSD	6	2	1	3	6	4	2	0	2	0
**Zn** (μg kg^–1^)	0	-	10.000	25.000	50 × 10^3^	100 × 10^3^	500 × 10^3^	1 × 10^6^	5 × 10^6^	10 × 10^6^
RSD	2.2	2	2.1	1.3	6.9	2.4	0.1	0.8	1.7	0.4

Three replicate samples, each weighing 0.1000 g (kernel, kernel shell, and flesh), were prepared. A mixture of 1.8 mL HNO_3_ (sp), 0.6 mL HCl (sp), and 0.6 mL H_2_O_2_ (sp) was added to PTFE-lined, sealed microwave vessels and allowed to react until gas evolution ceased. After digestion under optimized microwave conditions (temperature (tp), time (tm), and power (pw)), the samples were cooled to room temperature (25 °C). The digested solutions were transferred into 15 mL Falcon tubes. The vessels were rinsed with 2 mL of 2 M HNO_3_ to prevent analyte loss. The final volume was adjusted by adding ultrapure water to a total volume of 15 mL, and the solutions were analyzed by ICP-MS.

### 2.5 Quality Control and Assurance

For microwave-assisted digestion, three replicate samples (0.1000 g each) were analyzed for each apricot component (kernels, shells, and flesh). Each digest was measured in triplicate by ICP-MS, and the mean values and standard deviations were calculated. The MWOD optimization parameters were selected based on the systematic variation of temperature, time, and power conditions reported in the literature. For each element, 36 measurements were performed for temperature, time, and power optimization, thereby increasing statistical reliability. Quality control procedures demonstrated that the results were both precise and accurate, supported by an increased measurement frequency and statistical confidence. Limits of detection (LODs) for all elements were calculated based on standard deviation and mean values.

For the determination of As, Hg, Cd, Pb, Se, Cu, and Zn in apricot kernel, kernel shell, and fruit pulp samples, a European Union Reference Bureau (BCR-414) certified plankton reference material was used (Supporting Information 4). The concentrations and measurements of these elements are presented in Table 3, and the accuracy of the method was confirmed by the analysis of the plankton reference material. The concentrations of As, Hg, Cd, Pb, Se, Cu, and Zn in the blank samples used during the analysis were determined as follows: <0.000 μg kg^–1^, 0.0193 μg kg^–1^, <0.000 μg kg^–1^, <0.000 μg kg^–1^, <0.000 μg kg^–1^, <0.000 μg kg^–1^, and 29.782 μg kg^–1^, respectively (Supporting Information 3).

**Table 3:** Element Concentrations in the Plankton Reference Material and in the Samples after Microwave Digestion

Elements	CRM (mg kg^–1^)	1st MWOD (mg kg^–1^)	Conv p.tage (%)	2nd MWOD (mg kg^–1^)	Conv p.tage (%)	3rd MWOD (mg kg^–1^)	Conv p.tage (%)
^a^ ^a^As	6.82 ± 0.28	7.31 ± 0.01	107.25	6.58 ± 0.02	96.55	6.39 ± 0.03	93.63
^a^Cd	0.383 ± 0.014	0.39 ± 0.003	103.06	0.40 ± 0.04	104.71	0.426 ± 0.04	111.26
^a^Hg	0.276 ± 0.018	0.261 ± 0.004	94.86	0.274 ± 0.03	99.30	0.266 ± 0.02	96.49
^a^Se	1.75 ± 0.10	1.64 ± 0.05	93.6	1.84 ± 0.00	105.65	1.736 ± 0.06	99.20
^a^Cu	29.5 ± 1.3	28.15 ± 0.08	95.44	28.55 ± 0.09	96.79	28.70 ± 0.09	97.27
^a^Zn	111.6 ± 2.5	121.35 ± 0.05	108.74	115.54 ± 0.05	115.54	112.80 ± 0.05	101.07
^a^Pb	3.97 ± 0.19	3.93 ± 0.02	98.96	4.16 ± 0.06	104.9	4.17 ± 0.06	104.91
^b^V	8.1 ± 0.18	8.20 ± 0.03	101.23	8.53 ± 0.24	105.41	7.94 ± 0.09	98.03
^b^Cr	23.8 ± 1.2	23.45 ± 0.19	98.55	26.73 ± 0.11	112.33	24.57 ± 0.25	103.24
^b^Mn	299 ± 13	304.92 ± 0.27	101.97	304.77 ± 0.27	101.93	305.34 ± 0.76	102.12
^b^Ni	18.8 ± 0.8	19.41 ± 0.10	103.28	19.91 ± 0.17	105.95	19.13 ± 0.10	101.76

a Elements analyzed in apricot samples and in the plankton reference material.

b Elements were analyzed only in the plankton reference material.

The blank solutions were analyzed by ICP-MS after every ten measurements to confirm the absence of contamination and ensure the accuracy of the measurements (QC). The calibration curves showed excellent linearity with correlation coefficients in the range of R = 0.9997–1.0000 (Appendix-2). The detection limits for As, Hg, Cd, Pb, Se, Cu, and Zn were 0.05377, 0.009159, 0.3623, 1.068, 0.08234, 0.03454, and 4.562 μg kg^–1^, respectively.

### 2.6 Statistical Analysis

Analysis of variance (ANOVA) was used to compare Cu, Zn, As, Se, Cd, Hg, and Pb element levels among different groups (core-K, core–shell-S, and meat-F). Normality and homogeneity of variance tests were conducted prior to analysis to verify parametric test assumptions. Pearson correlation analysis was performed to evaluate relationships among elements.^61,62^

## 3 Results and Discussion

After separation and drying of apricot kernel, kernel shell, and flesh samples, comprehensive results were obtained through microwave digestion and ICP-MS analysis. Lead (Pb), cadmium (Cd), mercury (Hg), and arsenic (As) are classified as priority toxic elements in international food safety frameworks due to their cumulative toxicity and adverse health effects, even at low exposure levels.^1,63^ In contrast, copper (Cu), zinc (Zn), and selenium (Se) are essential trace elements with important physiological roles but may pose health risks when intake exceeds recommended limits. Therefore, monitoring both deficiency and excess levels is critical.^63^ The simultaneous evaluation of toxic and essential elements in apricot matrices provides a comprehensive assessment of dietary exposure and potential health risks in line with established food safety guidelines.

In the literature, studies using ICP-MS have included: a. Solvent method: nitric acid+hydrochloric acid+hydrogen peroxide; b. Use of CRM 414 plankton material for verification. c. Different optimization applications: d. Statistical calculations and verification and evaluation methods.^18,21,22,26^ In addition to these established approaches, the present study was designed based on EPA Method 3052 and its analytical framework, ensuring methodological robustness and reliability.^57,58^

### 3.1 Optimization of Temperature Variation

Table 4 shows the experimental parameters used for temperature optimization. Figure 1 presents the ICP-MS measurement results of toxic elements in apricot kernel, kernel shell, and flesh samples after microwave digestion at 180 °C, 200 °C, and 220 °C. The highest values obtained from ICP-MS measurements under these digestion conditions were as follows: Cu: 0.357 mg kg^–1^, Zn: 6.65 mg kg^–1^, As: 10.76 μg kg^–1^, Se: 8.66 μg kg^–1^, Cd: 21.13 μg kg^–1^, Hg: 0.45 μg kg^–1^, and Pb: 1.45 mg kg^–1^. When compared with World Health Organization (WHO) concentration limits (Cw), Cu, Zn, As, Cd, and Hg exceeded the reference values, whereas Se and Pb remained below the limits.^14^ The lowest measured concentrations were 0.08 mg kg^–1^ (Cu), 0.13 mg kg^–1^ (Zn), 0.14 μg kg^–1^ (As), 0.08 μg kg^–1^ (Se), 1.01 μg kg^–1^ (Cd), 0.05 μg kg^–1^ (Hg), and 0.08 mg kg^–1^ (Pb), respectively.^14^ The results further indicated that the highest Zn concentration (2.278 mg kg^–1^) was observed in the flesh sample from Site I at 180 °C, whereas the highest Cd concentration (17.466 μg kg^–1^) was detected in the flesh sample from Site III (Supporting Information 2). Comparison with previously published data on cornflakes provides additional context for these findings.^54^ In that study, the reported concentrations of As (0.0831 μg kg^–1^), Cd (0.0636 μg kg^–1^), Hg (0.0233 μg kg^–1^), and Pb (0.0689 mg kg^–1^) were substantially lower than the maximum values obtained in the present study, particularly for Cd and Zn. These differences may be attributed to variations in geographical origin, soil–plant transfer mechanisms, agricultural practices, and analytical procedures, including digestion efficiency and matrix effects.

**Table 4:** Parameters of the Temperature Variation Optimization

Microwave Digestion Program			
Step (Process)	Temperature (°C)	Time (min)	Power (W)
I	180	15	800
II	200	15	800
III	220	15	800

**Figure 1:**
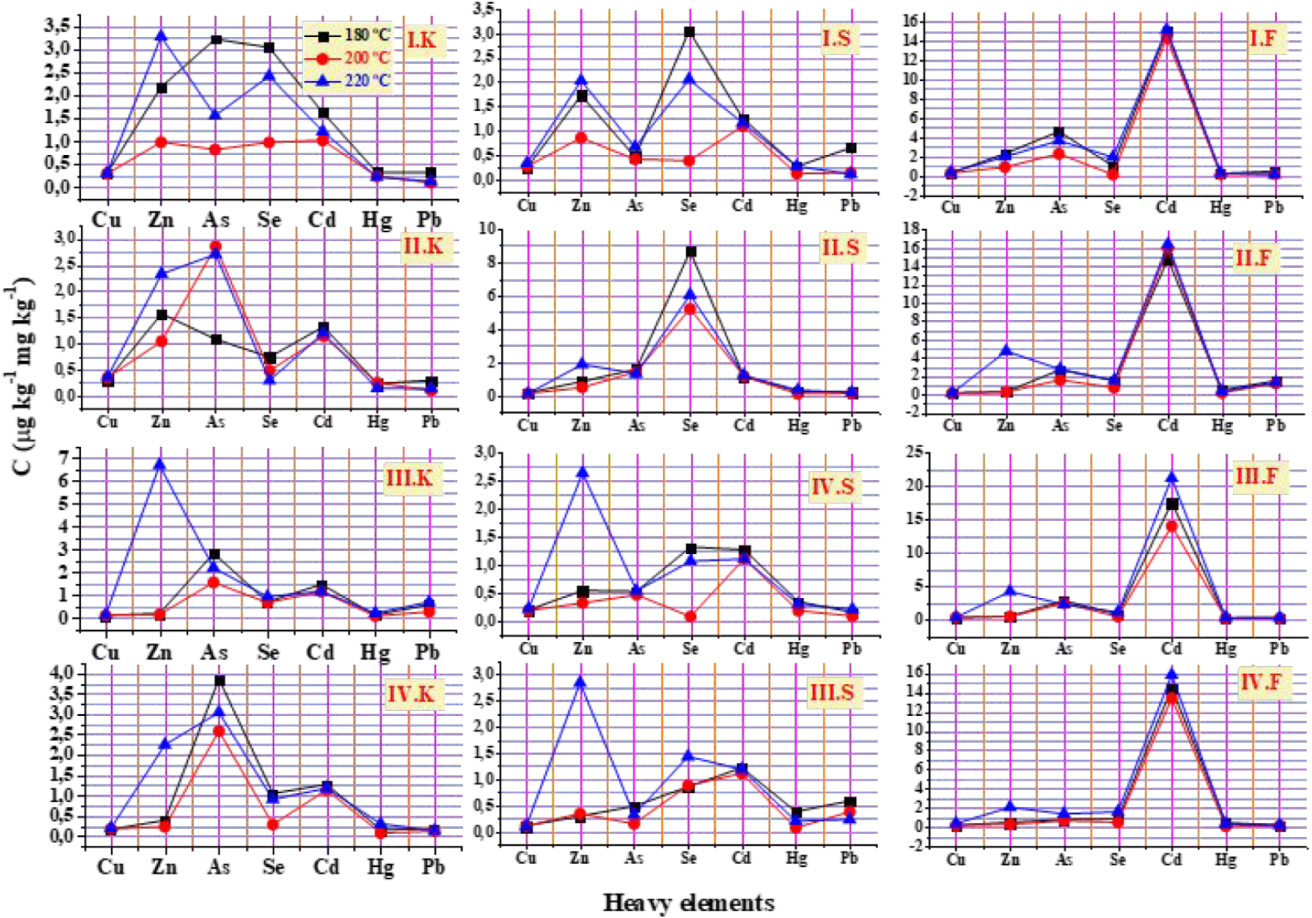
ICP-MS measurements of apricot’s kernel regions I.K, II.K, III.K, IV.K, kernel’s shell, I.S, II.S, III.S, IV.S, and flesh I.F, II.F, III.F, IV.F in a microwave oven (at 800 W for 15 min) at 180 °C, 200 °C, and 220 °C temperature variations (Cu, Zn, and Pb concentrations: C × 10^3^ μg kg^–1^).

Furthermore, the trace element levels determined in this study were compared with previously reported data for dried apricots from Türkiye. Overall, the elemental distribution pattern showed good agreement with the literature, especially for essential elements such as Fe, Zn, and Cu, which were within reported ranges. Observed quantitative differences may be associated with variations in soil composition, environmental conditions, cultivation techniques, and postharvest processing methods such as drying. These findings suggest that, despite some variability, the trace element profiles of the analyzed apricot samples are generally consistent with previously published studies. The results of microwave digestion at 200 °C (the second stage of temperature optimization) showed that the highest concentration of Pb (1.174 mg kg^–1^) was detected in the flesh sample from Site II, while the highest concentration of Cd (15.932 μg kg^–1^) was also observed in the flesh sample from Site II. Similarly, the results obtained at 220 °C (the third stage of temperature optimization) indicated that the highest concentration of Zn (6.650 mg kg^–1^) was found in the kernel sample from Site III, whereas the highest Cd concentration (21.127 μg kg^–1^) was detected in the flesh sample from Site III. Based on the μg kg^–1^-level measurements, it was observed that increasing temperature significantly enhanced the detection and extraction of cadmium.

An increase in temperature during microwave-assisted acid digestion improved the extraction efficiency of Zn and Pb, while the extraction efficiencies of As, Hg, Cd, Se, and Cu showed a decreasing trend. When these results were compared with World Health Organization (WHO) (2007) concentration limits (Cw), it was determined that Pb levels in Sites I, II, and III (flesh samples) and Se levels in the kernel from Site I exceeded recommended limits and may pose potential health risks.^1^

In this study, analysis of variance (ANOVA) was used to compare the elemental levels of Cu, Zn, As, Se, Cd, Hg, and Pb among different sample groups (kernel (K), kernel shell (S), and flesh (F)). Prior to analysis, normality and homogeneity of variance assumptions were tested. The sample size (*n* = 36) for temperature optimization was clearly defined. The Kolmogorov–Smirnov test indicated that the data were normally distributed, as all *p*-values were greater than 0.05. The assumption of homogeneity of variance was verified using Levene’s test, which also produced *p*-values above 0.05, confirming homogeneity. According to the results of one-way ANOVA, statistically significant differences were found among the groups for Zn (p = 0.000) and Hg (*p* = 0.000) (*p* < 0.05). No statistically significant differences were observed for the other elements (*p* > 0.05). Post hoc comparisons were performed using Duncan’s multiple range test, and the differences between groups are presented in the corresponding table (*p* < 0.05).

Table 5 presents the results of variance analysis for toxic elements in apricot kernel, kernel shell, and flesh samples following microwave digestion at 180 °C, 200 °C, and 220 °C. The results of Pearson’s correlation analysis, conducted to evaluate the relationships among elements under temperature optimization conditions, are presented below. According to the analysis, a statistically significant negative correlation was observed between Cu and Pb (r = −0.368, *p* < 0.05), indicating that an increase in Cu concentration was associated with a decrease in Pb concentration. No other statistically significant correlations were identified among the remaining element pairs (*p* > 0.05). Weak positive correlations were observed between Zn and Cu (r = 0.149) and between Zn and Pb (r = 0.149); however, these relationships were not statistically significant. Similarly, As showed weak positive correlations with Cu (r = 0.200), Zn (r = 0.162), and Pb (r = 0.143), but these correlations were not statistically significant.

**Table 5:** Results of the Variance Analysis of Temperature

	Cu	Zn	As	Se	Cd	Hg	Pb
K	0.19 ± 0.02	0.92 ± 0.23^a^	2.06 ± 0.42	2.49 ± 0.79	6.01 ± 2.01	0.26 ± 0.03^a^	0.43 ± 0.11
S	0.18 ± 0.03	0.5 ± 0.1^b^	1.41 ± 0.27	0.87 ± 0.4	5.53 ± 1.89	0.12 ± 0.02^b^	0.23 ± 0.09
F	0.22 ± 0.03	3.05 ± 0.42^a^	1.84 ± 0.31	1.75 ± 0.43	6.47 ± 2.3	0.24 ± 0.02^a^	0.28 ± 0.1
Sig.	0.602	0.000	0.390	0.144	0.950	0.000	0.365

a K, S, F in the same column indicate variation between groups.

b Ranging of temperature.

Se exhibited a negative correlation with Cd (r = −0.255) and a positive correlation with Hg (r = 0.217); however, both relationships were statistically insignificant. Cd showed a moderate positive correlation with As (r = 0.320), which was also not statistically significant. Furthermore, Hg demonstrated weak positive correlations with Pb (r = 0.300), Cd (r = 0.044), and Se (r = 0.217); however, none of these correlations reached statistical significance (*p* > 0.05). Overall, the limited number of significant correlations suggests that temperature variation during microwave digestion had a restricted influence on interelement relationships. However, the significant Cu–Pb relationship may indicate differences in elemental sources, mobility, or competitive behavior during extraction. These findings also suggest that temperature may have a partial additive effect on the extraction efficiency of certain elements, although this effect is not consistently reflected across all element pairs.^21^

Table 6 presents the Pearson correlation analysis results for toxic elements in apricot kernel, kernel shell, and flesh samples following microwave digestion at 180 °C, 200 °C, and 220 °C (Supporting Information 6). In the multielement analysis of red pepper conducted by TokalıoĞlu et al., strong positive correlations (*p* < 0.05) were observed among V, Cr, Cd, and Pb at the 99% confidence level, and between Fe and Zn at the 95% confidence level. In the same study, significant positive correlations were reported for Cu–Zn (r = 0.586), Zn–Pb (r = 0.507), Ni–As (r = 0.478), and As–Cd (r = 0.465) at the 99% confidence level.^21^

**Table 6:** Correlation Analysis of Temperature

	Cu	Zn	As	Se	Cd	Hg	Pb
Cu	1						
Zn	149	1					
As	200	162	1				
Se	008	055	–.116	1			
Cd	001	056	320	–.255	1		
Hg	169	195	–.040	217	044	1	
Pb	–.368	149	143	–.037	288	300	1

In contrast to these findings, the present study revealed that correlations among elements generally decreased with an increase in temperature, and only the negative correlation between Cu and Pb remained statistically significant. Correlations among other element pairs were weak and statistically insignificant, indicating that temperature variation had a limited influence on interelement relationships.

### 3.2 Optimization of Time Variation

The parameters for time optimization are presented in Table[Table tbl7] and Figure 2 illustrates the ICP-MS measurement results of toxic elements in apricot kernel, kernel shell, and flesh samples following microwave digestion at 10, 15, and 20 min. The highest concentrations obtained from ICP-MS measurements were Cu: 0.61 mg kg^–1^, Zn: 3.609 mg kg^–1^, As: 23.913 μg kg^–1^, Se: 11.528 μg kg^–1^, Cd: 6.194 μg kg^–1^, Hg: 2.778 μg kg^–1^, and Pb: 3.902 mg kg^–1^. When compared with WHO concentration limits (Cw), Cu, Zn, As, Cd, and Hg exceeded the reference values, whereas Se and Pb remained below the limits.^14^ The lowest measured values were 0.017 mg kg^–1^ (Cu), 0.034 mg kg^–1^ (Zn), 0.148 μg kg^–1^ (As), 0.027 μg kg^–1^ (Se), 0.754 μg kg^–1^ (Cd), 0.096 μg kg^–1^ (Hg), and 0.017 mg kg^–1^ (Pb), respectively.

**Table 7:** Parameters of the Time Variation Optimization

Process of Microwave-Assisted Acid Digestion					
	Temperature (°C)				
Step (Process)	Kernel	Shell	Flesh	Time (min)	Power (W)
I	180	180	220	10	800
II	180	180	220	15	800
III	180	180	220	20	800

**Figure 2:**
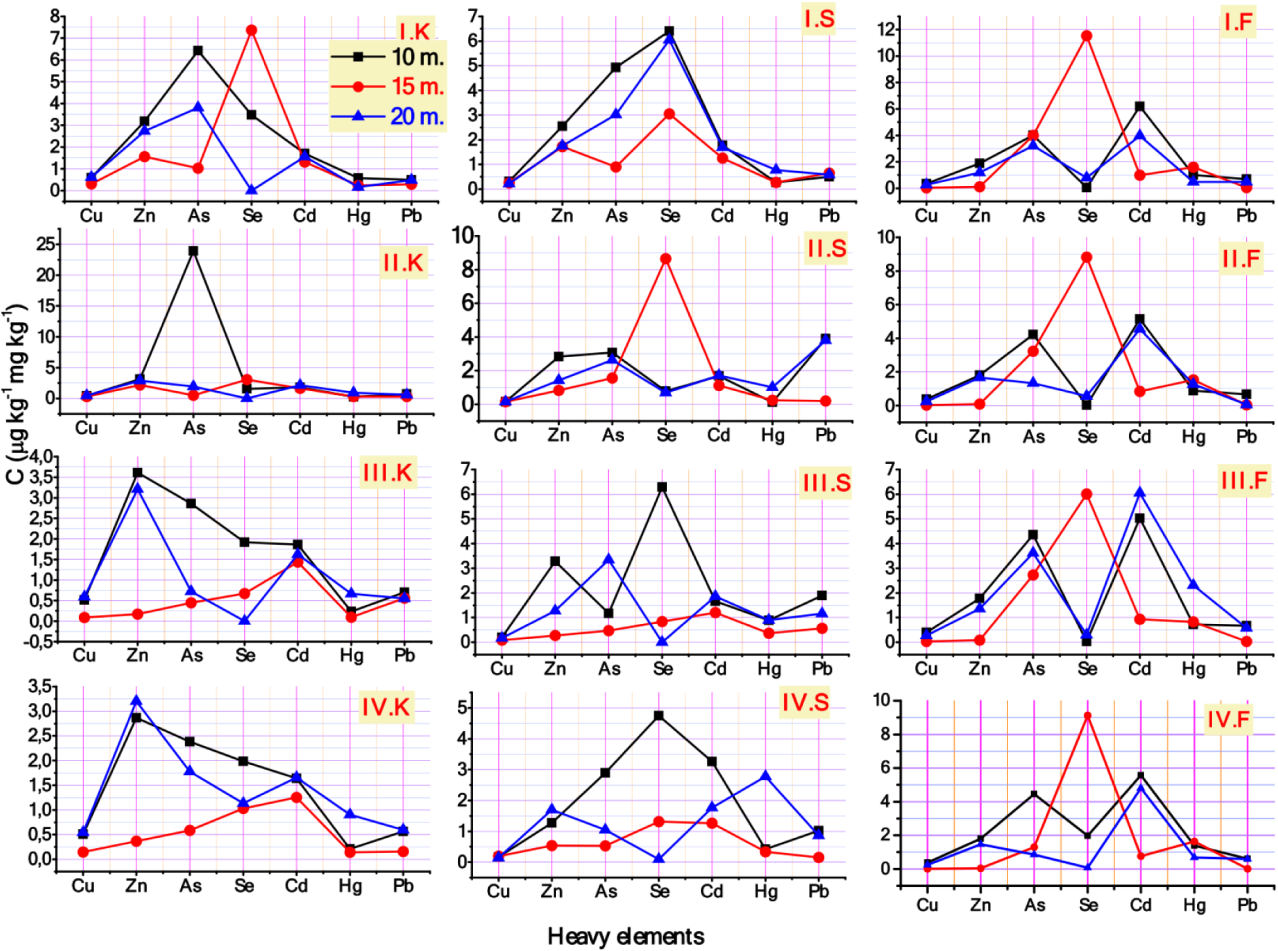
ICP-MS measurements of apricot’s kernel sites I.K, II.K, III.K, IV.K kernel’s shell, I.S, II.S, III.S, IV.S, and flesh I.F, II.F, III.F, IV.F in a microwave oven (at 180 °C and 800 W) at 10, 15, and 20 min (Cu, Zn, and Pb concentrations: C × 10^3^ μg kg^–1^).

During the first digestion step (10 min), the highest Pb concentration (3.902 mg kg^–1^) was observed in the kernel shell from Site II, while the highest As concentration (23.91 μg kg^–1^) was detected in the kernel from Site II. At 15 min, the highest Zn concentration (2.178 mg kg^–1^) was found in the kernel from Site II, whereas the highest Se concentration (11.528 μg kg^–1^) was observed in the flesh from Site I. At 20 min, the highest Pb concentration (3.802 mg kg^–1^) was again detected in the kernel shell from Site II, while the highest Se concentration (6.050 μg kg^–1^) was found in the shell from Site I.

The results indicate that increasing the digestion time enhanced the extraction efficiency of Se, reaching a maximum at 15 min, whereas no consistent effect was observed for other elements (As, Hg, Cd, Cu, and Pb). This behavior may be attributed to differences in the physicochemical composition of apricot components (kernel, shell, and flesh), as well as variations in the geological characteristics of the cultivation areas.^64^ When compared with WHO standards, it was determined that Pb concentrations in kernel shells from site II and Se concentrations in flesh samples from site I (at 15 min) exceeded recommended limits and may pose potential health risks.^14^

According to the results of the one-way analysis of variance (ANOVA) performed to compare heavy metal concentrations among different sample groups (kernel (K), kernel shell (S), and flesh (F)) under time optimization conditions, statistically significant differences were observed for Cu (*p* = 0.001), Zn (*p* = 0.000), As (*p* = 0.027), Se (*p* = 0.002), Cd (*p* = 0.004), and Pb (*p* = 0.046) (*p* < 0.05).

The sample size for time optimization was n = 36. The highest Cu (0.36 ± 0.04 mg kg^–1^), Zn (2.50 ± 0.22 mg kg^–1^), As (5.39 ± 1.73 μg kg^–1^), Cd (3.11 ± 0.53 μg kg^–1^), and Pb (1.04 ± 0.28 mg kg^–1^) concentrations were detected in the kernel (K) group. In contrast, the highest Se concentration (5.12 ± 1.13 μg kg^–1^) was observed in the kernel shell (S) group.

No statistically significant difference was found for Hg (p = 0.104), indicating similar concentration levels across all sample groups. Differences among groups were further supported by Duncan’s multiple range test, where distinct letter groupings indicated statistically significant differences (*p* < 0.05). These findings demonstrate that digestion time significantly affects the extraction efficiency of several elements. Table 8 presents the ANOVA results for toxic elements in apricot samples following microwave digestion at 10, 15, and 20 min (Supporting Information 6).

**Table 8:** Results of the Variance Analysis of Time

	Cu	Zn	As	Se	Cd	Hg	Pb
K	0.36 ± 0.04^a^	2.5 ± 0.22^a^	5.39 ± 1.73^a^	2.32 ± 0.68^b^	3.11 ± 0.53^a^	0.58 ± 0.12	1.04 ± 0.28^a^
S	0.13 ± 0.03^b^	0.66 ± 0.22^b^	1.44 ± 0.35^b^	5.12 ± 1.13^a^	1.16 ± 0.07^b^	0.63 ± 0.17	0.25 ± 0.07^a^
F	0.33 ± 0.05^a^	1.99 ± 0.23^a^	2.27 ± 0.33^b^	0.81 ± 0.49^b^	2.78 ± 0.46^a^	1.07 ± 0.22	0.91 ± 0.27^a^ ^b^
Sig.	0.001	0.000	0.027	0.002	0.004	0.104	0.046

a K, S, and F in the same column indicate variation between groups.

b Ranging of time.

Table 9 presents the results of Pearson correlation analysis conducted to evaluate relationships among elements under time optimization conditions. A strong and statistically significant positive correlation was observed between Cu and Zn (r = 0.822, *p* < 0.01), indicating that these elements tend to increase together. In contrast, a significant negative correlation was identified between Cu and Se (r = −0.454, *p* < 0.01), suggesting that an increase in Cu concentration is associated with a decrease in Se concentration. Similarly, Zn showed a significant negative correlation with Se (r = −0.342, *p* < 0.05), and Se exhibited a significant negative correlation with Cd (r = −0.480, *p* < 0.01). These findings indicate inverse relationships between these element pairs during time optimization. No statistically significant correlations were observed among As, Hg, and Pb (*p* > 0.05). A weak positive correlation was found between Hg and Cd (r = 0.291), but this relationship was not statistically significant. Similarly, Pb showed weak and insignificant correlations with all other elements. The strong correlations observed for Cu–Zn and Se–Cd suggest possible similarities in elemental sources, chemical behavior, or extraction mechanisms under varying digestion times. However, the absence of significant correlations among most elements indicates that time optimization has a selective rather than a uniform effect on interelement relationships. Table 9 summarizes the correlation coefficients for all element pairs under time optimization conditions.

**Table 9:** Correlation Analysis of Time

	Cu	Zn	As	Se	Cd	Hg	Pb
Cu	1						
Zn	822	1					
As	243	297	1				
Se	–.454	–.342	–.035	1			
Cd	238	096	087	–.480	1		
Hg	–.252	–.216	–.079	065	291	1	
Pb	–.074	300	030	–.274	016	–.059	1

Overall, statistically significant and strong correlations were observed for specific element pairs, particularly Cu–Zn and Se–Cd. These findings suggest that certain elements may exhibit coupled behavior during extraction and digestion processes. However, since most element pairs did not show statistically significant relationships, it can be concluded that time variation does not consistently influence interelement correlations, and some observed associations may result from random variability rather than systematic effects.

### 3.3 Optimization of Power Variation

Table 10 presents the parameters for power optimization. Figure 3 illustrates the ICP-MS measurement results of toxic elements in apricot kernel, kernel shell, and flesh samples following microwave digestion at 600, 800, and 1000 W.

**Table 10:** Parameters of the Power Variation Optimization

Process Steps of Microwave-Assisted Acid Digestion					
	Temperature (°C)				
Step (Process)	Kernel	Shell	Flesh	Time (min)	Power (W)
I	180	180	220	20	600
II	180	180	220	20	800
III	180	180	220	20	1000

**Figure 3:**
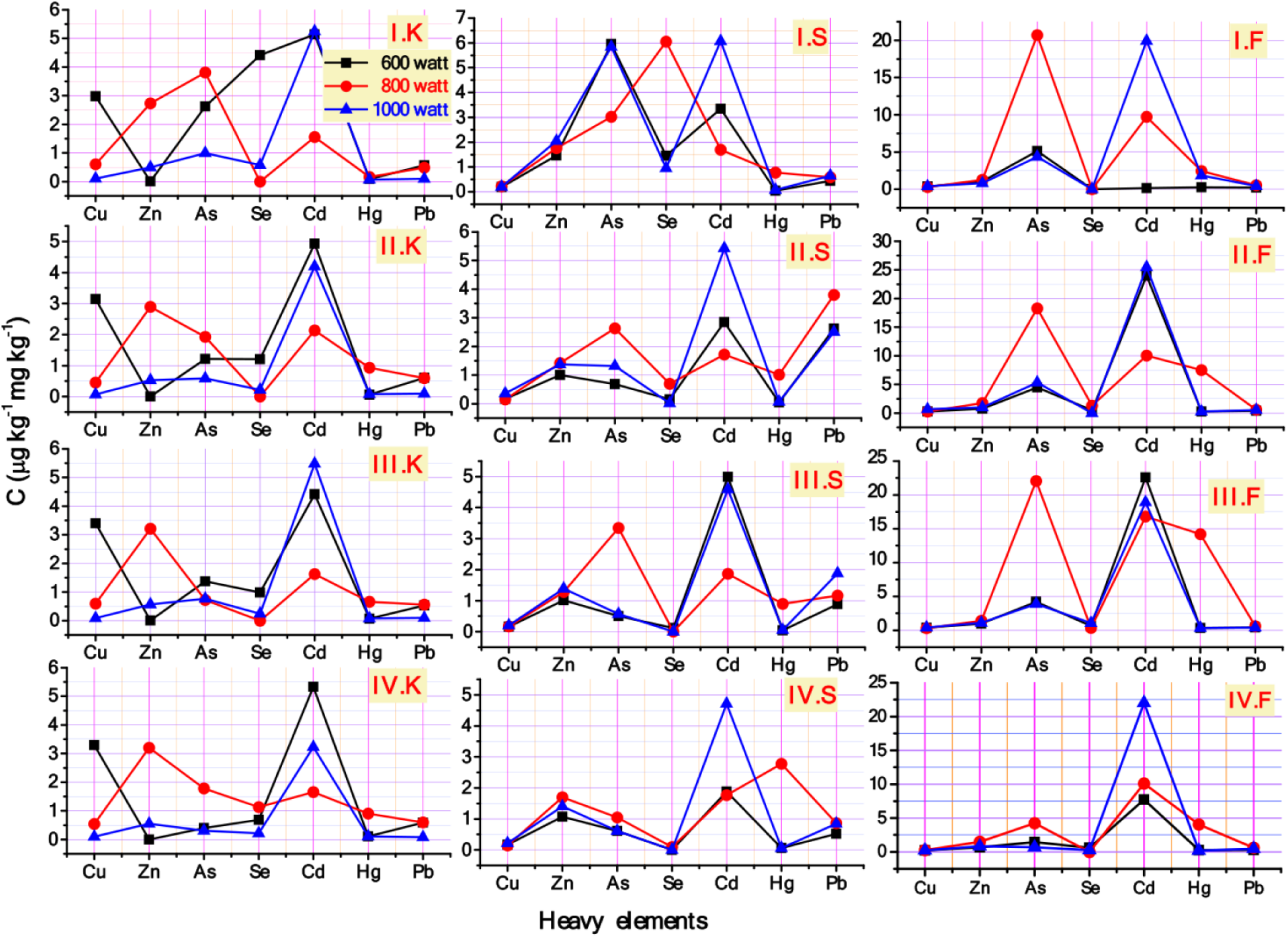
ICP-MS measurements apricot’s kernel sites I.K, II.K, III.K, IV.K kernel’s shell, I.S, II.S, III.S, IV.S, and flesh I.F, II.F, III.F, IV.F at microwave digestion at 600, 800, and 1000 W (180 °C for 20 min) (Cu, Zn, and Pb concentrations: C × 10^3^ μg kg^–1^).

During the first stage (600 W), the highest Cu concentration (3.402 mg kg^–1^) was observed in the kernel from Site III, while the highest Cd concentration (23.963 μg kg^–1^) was detected in the flesh from Site II. At 800 W, the highest Hg concentration (14.214 μg kg^–1^) was recorded in the flesh from Site III, and the highest As concentration (22.043 μg kg^–1^) was observed in the flesh samples from Sites I and III. At 1000 W, the highest Pb concentration (2.509 mg kg^–1^) was detected in the kernel shell from Site II, while the highest Cd concentration (25.491 μg kg^–1^) was found in the flesh from Site II.

These results indicate that increasing microwave power enhances the extraction efficiency of Cd, while As and Zn reach maximum extraction levels at 800 W. No consistent effect of power variation was observed for Hg, Cu, and Pb. This may be attributed to geological differences in cultivation areas and compositional differences among apricot tissues.^65^ When compared with WHO standards, it was determined that Pb concentrations in kernel shells (Sites I–III) and Hg concentrations in flesh samples (Sites II and III at 800 W) exceeded recommended limits, indicating potential health risks if consumed in excessive amounts.

In this study, detailed quantitative measurements of heavy metals in apricots, which have received geographical indication in IĞdır province and its districts and are among the major fruits in the food chain, were carried out by ICP-MS in microwave-assisted acid digestion, and the highest concentrations of selenium, cadmium, and mercury were found in the flesh. Sucharova and Suchara showed that the type of solvent-HNO_3_ + H_2_O_2_ (procedure A), HNO_3_ + H_2_O_2_ + HF + H_3_BO_3_ (procedure B), and HNO_3_ + H_2_O_2_ + HBF_4_ (procedure C)-should be taken into account for the identification of trace elements in leaves by ICP-MS using the MWOD procedure of several plant materials.^35^ In the present study, it was demonstrated that microwave-assisted acid digestion of apricot fruit had an effect on the type of elements, apricot cultivation area, and, most importantly, the quantitative amounts of toxic elements in the site where apricot is cultivated.^66^ Despite studies in which no direct correlation was established between the toxic elements and the geological characteristics of the sites where apricot ripes are cultivated, volcanic rocks in particular were found to produce toxic effects.^67^ In the present study, the toxic elements were determined in apricot ripe by ICP-MS after optimization of MWOD of apricot ripe. The highest concentrations of Cu, Zn, As, Se, Cd, Hg, and Pb were determined after obtaining the data.

The wet digestion method was used to determine K, Mg, Ca, Cu, Zn, Fe, and Mn in IĞdır apricot flesh by AAS, and the concentrations were found to be K: 262.20–144.86, Mg: 14.54–8.39, Ca: 13.66–7.53, Cu: 0.27–0.11, Zn: 0.15–0.06, Fe: 1.06–0.37, and Mn: 0.09–0.04 mg 100 g^–1^. In apricot kernels, these values were reported as K: 74.5–467.6, Mg: 164.53–126.86, Ca: 40.63–30.46, Cu: 1.51–1.05, Zn: 2.39–2.01, Fe: 2.91–2.11, and Mn: 0.53–0.36 mg 100 g^–1^, respectively.^43^ On the other hand, in the present study, more apricot samples were used to determine As, Cd, Se, Hg, and Pb, which were not studied in apricots and their kernels, and ICP-MS was used in the measurements.

In their mineral study, HacıseferoĞulları et al. determined K, P, Ca, Na, and Mg concentrations in different apricot fleshes using ICP-AES and the microwave wet digestion technique, but they did not study heavy metals.^11^ The concentrations of K, Na, Ca, Mg, P, Fe, Zn, Se, and Mn in apricot fleshes digested in a microwave were found to be 3219, 17.8, 233.7, 222.0, 237.9, 7.94, 4.24, 0.310, and 2.67 mg per 100 g^–1^, respectively, by AAS and ICP-MS. The concentrations of Zn and Se in the samples we studied were found in the range of 6.650–0.1293 mg kg^–1^, 7.366–0.264 μg kg^–1^ in the kernel, and 3.212–0.065 mg kg^–1^, 4.416–not detected in the flesh, respectively. This concentration difference was attributed to the cultivation area of apricots, the anthropogenic effect, and most importantly, the new application process.

The range of Zn concentration found in the kernel of Malatya apricot (Türkiye) (0.129–6.650 μg kg^–1^) in the present study compared to that of Alpaslan and Hayta in the determination of Na: 35.2–36.8, K: 473–570, Ca: 1.8–2.4, Mg: 113–290, Fe: 2.14–2.82, and Zn: 2.33–3.15 mg/100 g dry matter in the kernel of Malatya apricot (Türkiye) can be explained by the fact that we used a more detailed method in the present study and the cultivation regions of apricot are different.^66^ Tokman determined Cd, Cu, and Pb in apricot ripe in Afyonkarahisar province by wet digestion as 0.351–0.215, 0.621–0.505, and 0.067–0.027 mg·kg^–1^, respectively, whereas 0.005–0.001, 3.402–0.065, and 0.602–0.095 mg·kg^–1^ were determined in Şalak apricot in the present study.^67^ SaraçoĞlu et al., measured Cu, Zn, Se, Cd, and Pb ions as 0.92–6.49, 0.97–8.27, 0.32–0.64 μg g^–1^, 0.02–0.72, and 0.72–3.77 μg g^–1^, respectively, by AAS and graphite furnace atomic absorption spectrometer (GFAAS) in the dried flesh of Kayseri apricot (Türkiye).^68^ However, none of these elements were measured using only ICP-MS in the present study but also 3.402–0.065, 3.212–0.002 mg kg^–1^, 4.416-nd, 5.483–1.556 μg g^–1^, and 0.602–0.081 mg kg^–1^ were determined accurately and precisely in the kernel, shell, and flesh, respectively. These results indicate that ICP-MS and AAS measurements resulted from the method applied to apricot flesh (drying) and the geological characteristics of the apricot cultivation area. A study conducted by Zahoor et al., in Pakistan, determined Zn, Cd, and Pb by AAS at concentrations of 1.5–2.00, 0.21–0.09, and 1.61–1.66 mg kg^–1^, respectively, as a result of wet digestion.^69^ Therefore, previous similar studies support the differences between the quantitative results of the elements.

According to WHO guidelines, the maximum daily intake limits for Cu, Zn, Pb, As, and Cd are 30 mg/day, 12–15 mg/day, 210 μg/day, 150 μg/day, and 60 μg/day, respectively. In the present study, the highest concentrations of Cu, Zn, As, and Cd did not exceed these limits. However, excessive consumption of apricots may still pose potential health risks due to cumulative exposure.^14^ For Pb, Se, and Hg, the WHO limits are 210 μg/day, 7 μg/day, and 5 μg/day, respectively. The maximum concentrations of Pb, Se, and Hg observed in this study exceeded these limits, indicating potential health concerns.^14^

In this study, one-way ANOVA was performed to evaluate differences in element concentrations among sample groups (K, S, and F) under power optimization conditions (*n* = 36). Table 11 presents the results. Statistically significant differences were observed for Zn (*p* = 0.000), Cu (*p* = 0.015), As (*p* = 0.040), and Hg (*p* = 0.001) (*p* < 0.05). The highest Zn (2.01 ± 0.22) and Hg (1.96 ± 0.60) concentrations were detected in the kernel shell (S), while the highest Cu (1.23 ± 0.42) concentration was observed in the kernel (K). The highest As concentration (6.96 ± 2.36) was also found in the shell (S). No statistically significant differences were observed for Se (*p* = 0.448), Cd (*p* = 0.202), and Pb (*p* = 0.693), indicating similar concentrations across groups. These results suggest that microwave power significantly influences the extraction of certain elements, while others remain unaffected.

**Table 11:** Results of the Variance Analysis of Power

	Cu	Zn	As	Se	Cd	Hg	Pb
K	1.23 ± 0.42^a^	0.67 ± 0.15^b^	2.38 ± 0.58^b^	0.9 ± 0.35	7.27 ± 2.23	0.14 ± 0.03^b^	0.67 ± 0.18
S	0.33 ± 0.05^b^	2.01 ± 0.22^a^	6.96 ± 2.36^a^	0.8 ± 0.5	5.06 ± 1.5	1.96 ± 0.6^a^	0.91 ± 0.27
F	0.25 ± 0.05^b^	1.01 ± 0.14^b^	2.11 ± 0.61^b^	0.3 ± 0.11	10.44 ± 2.42	0.27 ± 0.15^b^	0.67 ± 0.22
Sig	0.015	0.000	0.040	0.448	0.202	0.001	0.693

a K, S, and F in the same column indicate variation between groups.

b Ranging of temperature.

The results of Pearson correlation analysis considering the power process were used to evaluate the correlations between the elements. According to the results of the analysis, there was a negative and statistically significant correlation between Zn and Cu (r = −0.448, *p* < 0.01). This result indicated that as the Cu concentration increased, there was a tendency for the Zn concentration to decrease. Furthermore, a positive and strong correlation was detected between As and Hg (r = 0.585, *p* < 0.01). As the correlations between other element pairs were analyzed, no significant correlation was detected, and their coefficients were generally low. A positive correlation with a decreasing tendency was demonstrated not only between Cu and Se (r = 0.302) but also between As and Cd (r = 0.308); however, these relationships were not statistically significant. There was also a negative correlation between Cd–Pb (r = −0.223) and Cd–Zn (r = −0.241), although these did not demonstrate significance. Table 12 confirmed the results of the Pearson correlation analysis performed to evaluate the correlations between the elements according to the power method (Supporting Information 6).

**Table 12:** Correlation Analysis of Power

	Cu	Zn	As	Se	Cd	Hg	Pb
Cu	1						
Zn	–.448	1					
As	–.144	133	1				
Se	302	–.096	009	1			
Cd	–.084	–.241	308	–.121	1		
Hg	–.171	241	585	–.005	102	1	
Pb	–.100	116	–.110	–.085	–.223	–.020	1

As a result of the power experiments, significant relationships were only determined between Cu–Zn and As–Hg pairs, while significant correlations were limited for other elements. However, in the multielement research of different brands of spices by TokalıoĞlu et al., significant correlations were found at the 95% confidence level between Cd–As (r = 0.519) and Pb–As (r = 0.614), Cd–As (r = 0.519), and Pb–Cd (r = 0.344).^44^ But this research indicates that, in the power process, relationships were detected between some elements, but correlations were generally low. The effect of the geological nature of the apricot cultivation areas, the parameters of the microwave oven, and the components that make up the apricot on the concentrations of heavy metal elements in apricot ripe was demonstrated.^64^ Furthermore, it was emphasized that these correlations not only represent a statistical relationship but also provide an environmental signal pointing to possible common sources; the practical importance of this for environmental monitoring, risk assessment, and pollution management was highlighted.

The MWOD studies conducted on apricots showed that Cd, Pb, and Se levels exceeded WHO limits for food consumption among the analyzed elements (Cu, Zn, As, Se, Cd, Hg, and Pb). In addition, other elements may accumulate in the human body and cause toxic effects when daily intake levels are exceeded.^14^ With these adjustments, the findings of the study are presented in a more holistic manner both statistically and environmentally.

### 3.4 Quality Assurance of Measurement Limits

Although apricot kernel, kernel shell, and fruit pulp samples were collected from four different regions, microwave-assisted digestion optimization procedures (temperature, time, and power) were repeated three times. This resulted in a total of 108 digestion and ICP-MS measurements for temperature optimization and 72 MWOD applications each for time and power optimization.

The accuracy and precision of the measurements were verified by

(i) Analysis of Blank Solutions and Certified Reference Material (BCR-414 plankton)
(ii) Statistical validation was done using SPSS-based ANOVA.

The relative standard deviation (RSD) values obtained are presented in Table 13.

**Table 13:** Statistical Deviation Limit Values (RSD) of ICP-MS Results

Elemt	Temp.	Time	Power	Cal	Ref	Cont/Blank spike
63 Cu	0.12–2.99	0.28–8.23	0.06–54.81	0–6	1.01–2.93	0.4–9.9
66 Zn	0.18–4.98	0.24–7.09	0.17–20.05	0.1–6.9	0.61–4.26	0.60–14.10
75 As	0.76–27.84	3.22–64.61	0.76–37.77	0.2–16	1.21–11.90	2.16–20.80
78 Se	4.38–191.23	8.08–270	13.84–278.25	0.4–40	0.01–71.19	6.00–49.50
111 Cd	0.15–25.55	0.42–8.31	3.15–207.28	1–6	15.81–39.18	3.73–21.2
201 Hg	1.29–36.09	1.46–18.70	0.95–17.24	0.8–14	65.73–327.87	3.33–11.9
208 Pb	0.10–29.92	0.57–7.13	7.14–69.78	0–6	0.62–3.08	0.80–6.2

These statistical deviation limit values indicate that the MWOD methods are quite successful.^69^ In microwave oven acid digestion optimization methods (MWOD), RSD values are evaluated as RSD > 50.00 (bad), RSD = 10.00–49.99 (not good), RSD = 5.00–9.99 (good), and RSD = 0.00–4.99 (excellent).^69^ According to these results, the standard deviation percentage rate is relatively high, especially due to the volatilization of SeCl_4_ into the gas phase. Therefore, reducing the use of HCl in the microwave oven optimization acid digestion method for selenium can provide more sensitive and precise ICP-MS values. Although it dissolves quite well in aqua regia and passes into solution in the form of a doubly charged ion, due to its volatility, it forms ions in the form of HgCl_2_ at low pH during solution preparation and in the form of Hg_2_(NO_3_)_2_·2H_2_O in the singly charged ion form. The conversion of these ions to each other also results in a relatively high percentage variation in the determination of this element by ICP-MS.

Particularly, the Cu–Zn and As–Hg correlation relationships in three different parts of apricot fruit have been determined in some studies in the literature, although they vary according to food type. It is a fact that positive correlations mostly increase the additive effect.

The Food and Agriculture Organization (FAO), the Environmental Protection Agency (EPA), the Joint FAO/WHO Expert Committee on Food Additives (JECFA), the European Food Safety Authority (EFSA), and the World Health Organization (WHO) Codex Alimentarius have declared the upper limits for daily consumption and weekly intake of heavy metals when they accumulate in the organism.^49,50,57–59^ When the tolerable amount exceeds these upper limits, diseases such as various types of cancer, kidney failure, and slowing of mental activities occur along with toxic effects. The limit values considered toxic for human health regarding toxic elements in ICP-MS measurements of MWOD extraction solution in apricots are given in Table 14.

**Table 14:** Limit Values Considered Toxic for Human Health (mg kg^–1^)

Element Type	Daily Intake Rate	Tolerable Limit Weight	Accepting Other Institution	WHO Satd. Lim.(mg/day)
Cu	0.5	0.7–0.9	FAO	0.3
Zn	15	8–11	FAO	12–15
As	0.015	0.05–0.1	EFSA/FDA	0.150
Se	0.5	0.1–0.3	EPA	0.007
Cd	0.18	0.005–0.007	EU regulation 2021/1323	0.060
Hg	0.015	0.02–0.01	EU regulation 2018/73	0.005
Pb	0.025	0.4–0.5	EU regulation 2021/1317	0.210

In this article, when apricot fruit pulp and seeds are consumed, the maximum values were determined as follows: Pb: 3.902 mg kg^–1^, Zn: 3.609 mg kg^–1^, Cu: 0.61 mg kg^–1^, As: 23.91 μg kg^–1^, Se: 11.528 μg kg^–1^, Hg: 2.778 μg kg^–1^, and Cd: 6.194 μg kg^–1^. According to this order, Pb, Zn, and Cu can accumulate in the organism and create toxic effects.

### 3.5 Assessment of Health Risks

The average amount of toxic metals ingested daily from apricot consumption can be calculated according to the following formula, in line with the recommendations of the Food and Agriculture Organization of the United Nations (FAO).^59^ The Estimated Daily Intake (EDI) of toxic metals through apricot consumption can also be calculated using the same formula in accordance with FAO recommendations:^59^

$$\mathrm{EDI}=\frac{\mathrm{Mean concentration}\left({\mathrm{mg kg}}^{-1}\right)\times \mathrm{Daily intake}\left(\mathrm{kg per day}\right)}{\mathrm{Body weight}\left(\mathrm{kg}\right)}$$

In the study conducted by Hassan et al., the toxic metal ratios consumed by consumers through food were calculated as approximately I–57 g for daily consumers, II–1.0 g for regular consumers, and III–0.2 g for rare consumers. In the research conducted by Hassan et al. on corn flakes, although Pb, As, Cr, Hg, and Cd toxic elements were (*p* ≥ 0.05), statistically significant differences (*p* ≤ 0.05) were determined in branded products.^54^ In research conducted on corn, rice, and wheat regarding toxic elements, the As ratio was 1.48 mg kg^–1^, 0.98 mg kg^–1^, and 0.46 mg kg^–1^, respectively, which means approximately twice the exposure limit in cereals (0.5 mg kg^–1^).^71–73^

In this article, the health risk ratio given in the following equation was considered for Pb, Zn, Cu, As, Se, Hg, and Cd toxic elements in apricot kernel, kernel shell, and fruit pulp, which were investigated using the MWOD method under temperature, time, and power optimization, and the hazard quotient (HQ) was calculated for each element:

$$\mathrm{HQ}=\frac{\mathrm{EDI}\;\left(\mathrm{mg per kg per day}\right)}{\mathrm{TDI}\;\left(\mathrm{Tolerable daily intake}\right)}$$

The TDI and EDI ratios used here are based on the ratios accepted by the WHO Codex Alimentarius (2014), the World Food and Agriculture Organization (WHO-FAO), the American Environmental Protection Agency (EPA), the Food Additives Authority (EFSA/FDA), and the European Food Safety Authority (EFSA).^58,60,70,71^ The average, minimum, maximum, and standard deviation values of toxic elements obtained from MWOD temperature, time, and power optimization in apricots are presented in Table 15.

**Table 15:** Average, Min, Max, and Standard Deviation Values of Toxic Elements in MWOD Temperature, Time, and Power Optimization in Apricots

Element	Temperaturea^a^	Timeb^b^	Powerc^c^	Toler Limit	Daily Intake	EDI Temperature	EDI Time	EDI Power	HQ Temperature	HQ Time	HQ Power
Cu	0.195	0.274	0.603	0.5	0.9	0.002309	0.003245	0.007141	0.004618	0.006489	0.014282
Zn	1.487	1.718	1.227	15	11	215.2237	248.6579	177.5921	14.34825	16.57719	11.83947
As	0.001	0.003	0.001	0.015	0.1	1.32 × 10^–06^	3.95 × 10^–06^	1.32 × 10^–06^	8.77 × 10^–05^	0.000263	8.77 × 10^–05^
Se	0.001	0.002	0.0006	0.5	0.3	3.95 × 10^–06^	7.89 × 10^–06^	2.37 × 10^–06^	7.89 × 10^–06^	1.58 × 10^–05^	4.74 × 10^–06^
Cd	0.001	0.002	0.004	0.18	0.007	9.21 × 10^–08^	1.84 × 10^–07^	3.68 × 10^–07^	5.12 × 10^–07^	1.02 × 10^–06^	2.05 × 10^–06^
Hg	0.0002	0.761	0.0004	0.015	0.01	2.63 × 10^–08^	0.0001	5.26 × 10^–08^	1.75 × 10^–06^	0.006675	3.51 × 10^–06^
Pb	0.235	0.454	0.483	0.025	0.5	0.001546	0.002987	0.003178	0.061842	0.119474	0.127105

a Mean of temperature opt.

b Mean of time opt.

c Mean of power opt.

In previous studies on edible mushrooms, fish, wheat, rice, corn, and corn flakes, it is emphasized that the generally low amounts of toxic elements in foods, according to WHO data, do not trigger various diseases by accumulating in the organism.^42,47,48,54,60,72^ In this research, since the calculated HQ values were much lower than 1, it was determined that—as long as it does not accumulate in the human organism—it will not pose a health risk (Supporting Information 1). Detailed analyses of apricots (kernel, kernel shell, and flesh) revealed that the maximum Zn value was 16.57, while the minimum Cd value was 1.02 × 10^–06^, as shown in Table 15. This means that the toxic effects of Zn on human health can only be observed when its HQ coefficient exceeds 15 mg kg^–1^ in a 70 kg person.

## 4 Conclusion

Apricot kernel, kernel shell, and flesh samples collected from four different regions were subjected to digestion processes that were repeated three times. To determine toxic elements (Cu, Zn, As, Se, Cd, Hg, and Pb), a total of 108 ICP-MS measurements were performed for temperature optimization during microwave-assisted acid digestion: 36 from the kernel, 36 from the kernel shell, and 36 from pulp samples. Similarly, 108 ICP-MS measurements were performed for time optimization and another 108 for power optimization with the same sample distribution. As part of the basic optimization grouping, quality control (QC) and quality assurance (QA) results for toxic elements in the ripe apricot were evaluated by using relative standard deviation (RSD) values obtained by ICP-MS. Although the 36 samples in each optimization group came from four different regions, the microwave digestion processes for temperature, time, and power were each repeated three times, increasing the statistical confidence interval for each element and ensuring reliable and accurate results.

This study demonstrates the importance of statistically supported optimization in microwave-assisted acid extraction for determining the elemental composition of the food samples. Analysis of apricot samples also shows that, although the daily dietary intake hazard quotient (HQ) values of toxic elements are not considered dangerous to human health, their accumulation in the body over time can contribute to the development of serious and potentially fatal diseases.

The presence of high concentrations of toxic metals in fish is of particular importance in relation to the FAO/WHO standards for Pb and Cd. The maximum permissible doses for an adult are 3 mg of Pb and 0.5 mg of Cd per week, but the recommended doses are only one-fifth of those quantities. Lead is known to induce reduced cognitive development and intellectual performance in children, as well as increased blood pressure and cardiovascular disease in adults.

The results demonstrated that heavy metal concentrations in apricot fruit are significantly influenced by the geological characteristics of cultivation areas, microwave digestion parameters, and the compositional structure of the fruit. Statistical analysis of microwave-assisted digestion processes (temperature, time, and power) further supported significant correlations among the Cu–Zn, As–Hg, and Se–Cd element pairs.

## Supplementary Material













## Notes

Health risk assessment based on ICP-MS results (XLSX)
Temperature optimization of ICP-MS (XLSX)
Plankton optimization of ICP-MS (XLSX)
Plankton of analysis (PDF)
ICP-MS calibration (Hg standard) (PDF)
Optimization of ICP-MS (XLSX)
