# Supplementary material for: The Effect of Microwave Oven Extraction Temperature, Time, and Power Optimization on the Determination of Heavy Metals in Apricot by ICP-MS
Source: ACS Omega. 2026 Jun 18;11(25):36454–67. doi: 10.1021/acsomega.5c13020 (PMC13325156; doi:10.1021/acsomega.5c13020)
Supplement: Supplementary file 4 [file ao5c13020_si_004.pdf]

European Commission

JOINT RESEARCH CENTRE
Directorate F – Health, Consumers and Reference Materials

CERTIFIED REFERENCE MATERIAL BCR® – 414

CERTIFICATE OF ANALYSIS

PLANKTON

Element	Mass fraction based on dry mass Certified value [1] [mg/kg]	Uncertainty [2] [mg/kg]	Number of accepted sets of results p
As	6.82	0.28	12
Cd	0.383	0.014	9
Cr	23.8	1.2	7
Cu	29.5	1.3	17
Hg	0.276	0.018	7
Mn	299	13	9
Ni	18.8	0.8	8
Pb	3.97	0.19	10
Se	1.75	0.10	8
V	8.10	0.18	4
Zn	111.6	2.5	15

1) Unweighted mean value of the means of p accepted sets of data, each set being obtained in a different laboratory and/or with a different method of determination. The certified values are traceable to the SI.

2) Half-width of the 95 % confidence intervals.

This certificate is valid for one year after purchase.

Sales date: 10.11.2025

The minimum amount of sample to be used is 100 mg.

NOTE

This material has been certified by BCR (Community Bureau of Reference, the former reference materials programme of the European Commission). The certificate has been revised under the responsibility of the European Commission's Joint Research Centre (JRC).

Brussels, March 1992
Latest revision: June 2017

Signed:

Dr. Doris Florian, Head of Unit
European Commission, Joint Research Centre
Directorate F – Health, Consumers and Reference Materials
Retieseweg 111
2440 Geel, Belgium

Indicative Values			
Element	Mass fraction		
	Indicative value[1)]	Uncertainty [2)]	Unit
Co	1.43	0.06	mg/kg
K	7.55	0.17	g/kg
Fe	1.85	0.19	g/kg
Mo	1.35	0.20	mg/kg
Sc	0.54	0.02	mg/kg
Sr	261	25	mg/kg

1) Mean value
2) Standard deviation

DESCRIPTION OF THE SAMPLE

The sample consists of a powder of freeze-dried plankton in a glass bottle. The bottle contains about 5 g of powder and a small PTFE ball which has been added to facilitate the homogenisation prior to use. Additional information on the preparation, the certified and indicative values is given in the certification report.

ANALYTICAL METHOD USED FOR CERTIFICATION

- Cold vapour atomic absorption spectrometry
- Cathodic stripping voltammetry
- Direct current plasma atomic emission spectrometry
- Differential pulse anodic stripping voltammetry
- Differential pulse cathodic stripping voltammetry
- Electrothermal atomic absorption spectrometry
- Electrothermal atomic absorption spectrometry with Zeeman background correction
- Flame atomic absorption spectrometry
- Hydride generation atomic absorption spectrometry
- Hydride generation inductively coupled plasma atomic emission spectrometry
- Inductively coupled plasma atomic emission spectrometry
- Inductively coupled plasma mass spectrometry
- Instrumental neutron activation analysis
- Isotope dilution mass spectrometry
- Mass spectrometry
- Neutron activation analysis with radiochemical separation
- Visible light or UV spectrometry

PARTICIPANTS

- Aristotelian University, Lab. Anal. Chemistry, Thessaloniki (GR)
- Danish Isotope Centre, Copenhagen (DK)
- E.C.N., Energieonderzoek Centrum Nederland, Petten (NL)
- Forschungszentrum für Umwelt und Gesundheit, Neuherberg (DE)
- Istituto Superiore di Sanità, Roma (IT)
- Instituut voor Nucleaire Wetenschappen, Rijksuniversiteit Gent (BE)
- European Commission, Joint Research Centre, Environment Institute, Ispra (IT)
- Kernforschungsanlage, Jülich (DE)
- Labor für Spurenanalytik, Bonn (DE)
- National Food Administration, Uppsala (SE)
- NLR “Demokritos”, Agia Paraskevi Attikis (GR)
- Presidio Multizonale di Prevenzione, Venezia (IT)
- Risø National Laboratory, Roskilde (DK)
- Universidad Complutense, Faculdad de Quimica, Madrid (ES)
- Università di Pavia, Chimica Generale, Pavia (IT)
- Universitaire Instelling Antwerpen, Wilrijk (BE)

SAFETY INFORMATION

The usual laboratory safety precautions apply.

INSTRUCTIONS FOR USE

The sample should be used as it is from the bottle. Before a bottle is opened, it should be shaken manually for 5 min so that the material is re-homogenised.
The correction to dry mass should be made on a separate portion of 100 mg which should be dried in an oven at 102 °C for 3-4 h until constant mass is attained (successive weighing should not differ by more than 0.2 mg). Dispose in accordance with good laboratory practice.

STORAGE

The tightly closed bottles may be kept at 18 ± 5 °C in the dark. The material picks up moisture when in prolonged contact with humid air.
However, the European Commission cannot be held responsible for changes that happen during storage of the material at the customer's premises, especially of opened samples.

LEGAL NOTICE

Neither the European Commission, its contractors nor any person acting on their behalf:

(a) make any warranty or representation, express or implied, that the use of any information, material, apparatus, method or process disclosed in this document does not infringe any privately owned intellectual property rights; or

(b) assume any liability with respect to, or for damages resulting from, the use of any information, material, apparatus, method or process disclosed in this document save for loss or damage arising solely and directly from the negligence of the European Commission's Joint Research Centre.

NOTE

A technical report on the production of BCR-414 is available on https://crm.jrc.ec.europa.eu A paper copy can be obtained from the JRC on request.

European Commission – Joint Research Centre,
Directorate F – Health, Consumers and Reference Materials
Retieseweg 111, B - 2440 Geel (Belgium)
Telephone: +32-(0)14-571.705 - Fax: +32-(0)14-590.406
