# Supplementary material for: The Effect of Microwave Oven Extraction Temperature, Time, and Power Optimization on the Determination of Heavy Metals in Apricot by ICP-MS
Source: ACS Omega. 2026 Jun 18;11(25):36454–67. doi: 10.1021/acsomega.5c13020 (PMC13325156; doi:10.1021/acsomega.5c13020)
Supplement: Supplementary file 5 [file ao5c13020_si_005.pdf]

Calibration for 009CALS.d

Batch Folder: D:\ICPMS\data\SERVETASKIN-180422.b\
Analysis File: SERVETASKIN-180422.batch.bin
DA Date-Time: 2022-04-20 09:07:40
Calibration Title:
Calibration Method: External Calibration
VIS Interpolation Fit:

Level	Standard Data File	Sample Name	Acq. Date-Time
1	008CALB.d	%1-HNO3-AQ	2022-04-19 13:24:35
2	009CALS.d	A-STD-0.5ppb+Hg	2022-04-19 13:31:18
3	012CALS.d	A-STD-1ppb+Hg	2022-04-19 13:51:29
4	015CALS.d	A-STD-2.5ppb+Hg	2022-04-19 14:11:20
5	018CALS.d	A-STD-5ppb+Hg	2022-04-19 14:31:08
6	021CALS.d	A-STD-10ppb+Hg	2022-04-19 14:50:57
7	022CALS.d	A-STD-50ppb	2022-04-19 14:57:32
8	023CALS.d	A-STD-100ppb	2022-04-19 15:04:03
9	024CALS.d	A-STD-500ppb	2022-04-19 15:10:31
10	025CALS.d	A-STD-1000ppb	2022-04-19 15:16:56

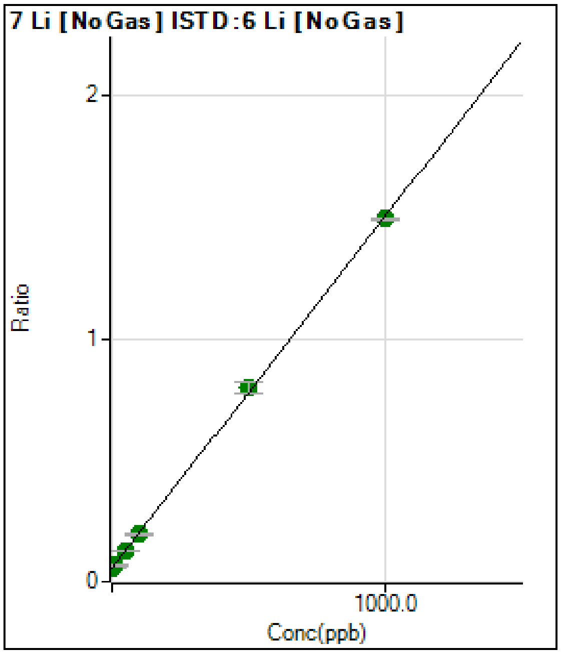

	Rjct	Conc.	Calc Conc.	CPS	Ratio	Det.	RSD
1	☐	0.000	0.000	1177920.24	0.0563	A	0.4
2	☑	0.500		1201278.81	0.0567	A	1.5
3	☑	1.000		1247495.94	0.0576	A	1.9
4	☑	2.500		1332392.51	0.0610	A	1.1
5	☐	5.000	4.643	1418490.29	0.0630	A	0.5
6	☐	10.000	7.959	1537368.09	0.0678	A	2.0
7	☐	50.000	49.543	2857460.93	0.1279	A	0.5
8	☐	100.000	96.032	4401878.02	0.1951	A	1.6
9	☐	500.000	513.702	16988130.72	0.7985	A	5.2
10	☐	1000.000	993.591	31620833.14	1.4918	A	0.3

y = 0.0014 * x + 0.0563
R = 0.9999
DL = 0.4167
BEC = 38.99

Weight: <None>
Min Conc: 0

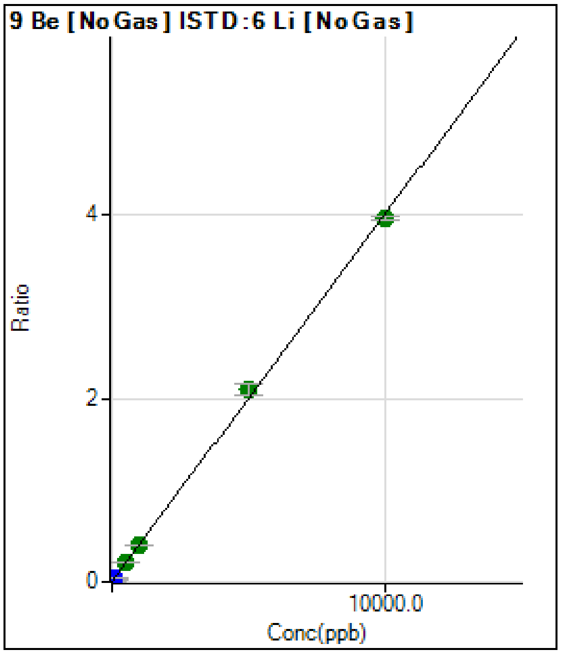

	Rjct	Conc.	Calc Conc.	CPS	Ratio	Det.	RSD
1	☐	0.000	0.000	188.90	0.0000	P	8.8
2	☐	5.000	7.029	59908.32	0.0028	P	0.7
3	☐	10.000	11.822	102814.04	0.0047	P	1.3
4	☐	25.000	22.584	197967.76	0.0091	P	3.1
5	☐	50.000	46.394	418571.83	0.0186	P	1.2
6	☐	100.000	82.822	752526.56	0.0332	P	0.6
7	☐	500.000	502.334	4497501.50	0.2013	A	0.4
8	☐	1000.000	998.043	9025232.16	0.4000	A	0.6
9	☐	5000.000	5249.681	44752907.11	2.1038	A	5.7
10	☐	10000.000	9875.431	83884072.64	3.9576	A	0.9

y = 4.0075E-004 * x + 9.0336E-006
R = 0.9996
DL = 0.005932
BEC = 0.02254

Weight: <None>
Min Conc: 0

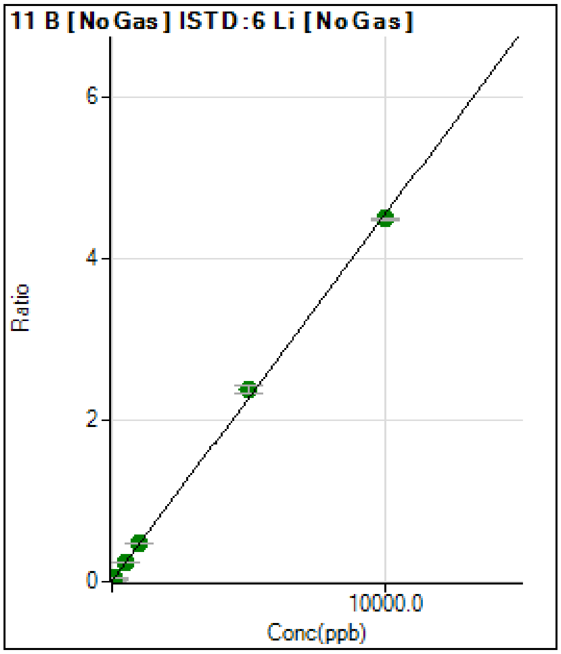

	Rjct	Conc.	Calc Conc.	CPS	Ratio	Det.	RSD
1	☐	0.000	0.000	183492.62	0.0088	P	0.6
2	☑	5.000		307465.12	0.0145	P	0.5
3	☑	10.000		353248.82	0.0163	P	1.8
4	☑	25.000		485927.17	0.0222	P	2.1
5	☐	50.000	50.013	707531.38	0.0314	P	1.5
6	☐	100.000	94.402	1168786.80	0.0516	A	0.3
7	☐	500.000	507.957	5339612.00	0.2390	A	0.8
8	☐	1000.000	1011.039	10538709.29	0.4670	A	0.5
9	☐	5000.000	5229.119	50617540.36	2.3790	A	4.7
10	☐	10000.000	9883.995	95146188.58	4.4889	A	0.3

y = 4.5327E-004 * x + 0.0088
R = 0.9996
DL = 0.3755
BEC = 19.36

Weight: <None>
Min Conc: 0

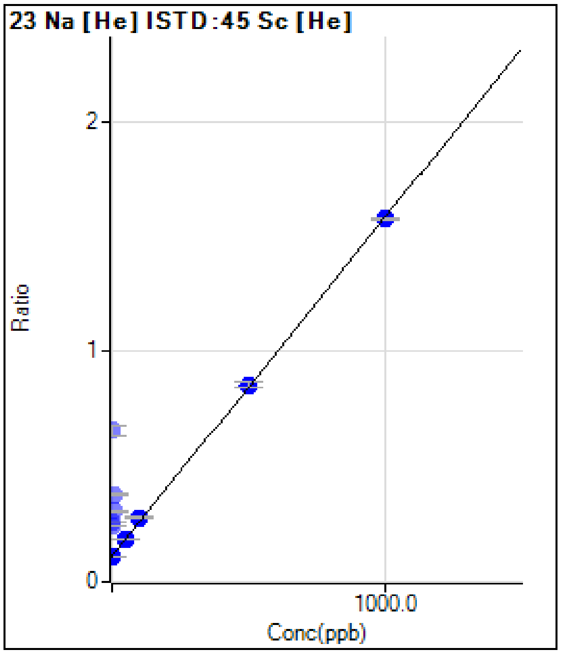

	Rjct	Conc.	Calc Conc.	CPS	Ratio	Det.	RSD
1	☐	0.000	0.000	21813.39	0.1109	P	0.5
2	☑	0.500		51095.94	0.2553	P	0.3
3	☑	1.000		48128.14	0.2420	P	1.0
4	☑	2.500		129062.03	0.6564	P	5.3
5	☑	5.000		77076.23	0.3798	P	1.3
6	☑	10.000		62681.90	0.3056	P	1.1
7	☐	50.000	49.050	37016.31	0.1830	P	0.5
8	☐	100.000	114.713	56385.93	0.2796	P	3.1
9	☐	500.000	505.269	163685.29	0.8542	P	2.6
10	☐	1000.000	995.942	291559.41	1.5761	P	0.9

y = 0.0015 * x + 0.1109
R = 0.9999
DL = 1.206
BEC = 75.36

Weight: <None>
Min Conc: 0

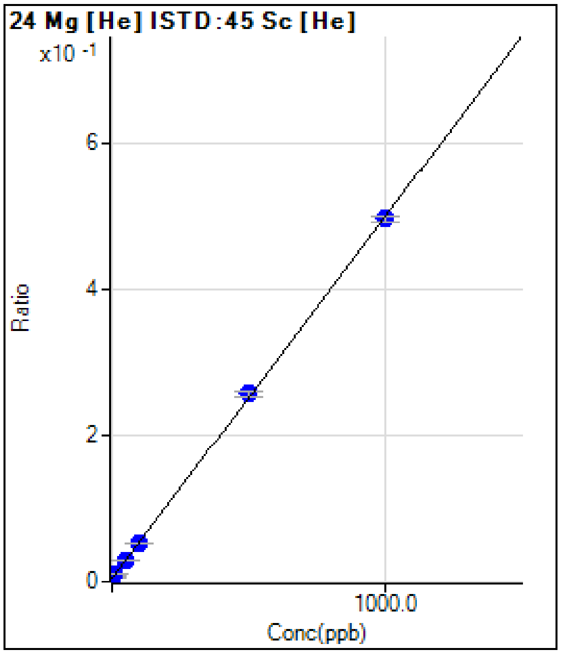

	Rjct	Conc.	Calc Conc.	CPS	Ratio	Det.	RSD
1	☐	0.000	0.000	870.06	0.0044	P	4.3
2	☑	0.500		1393.45	0.0070	P	2.0
3	☑	1.000		1615.70	0.0081	P	2.7
4	☑	2.500		2069.09	0.0105	P	10.1
5	☑	5.000		2164.66	0.0107	P	6.0
6	☐	10.000	10.283	1950.22	0.0095	P	2.5
7	☐	50.000	48.876	5783.33	0.0286	P	3.7
8	☐	100.000	99.065	10773.48	0.0534	P	0.3
9	☐	500.000	511.124	49292.18	0.2573	P	3.3
10	☐	1000.000	994.585	91824.78	0.4964	P	1.7

y = 4.9466E-004 * x + 0.0044
R = 0.9999
DL = 1.146
BEC = 8.94

Weight: <None>
Min Conc: 0

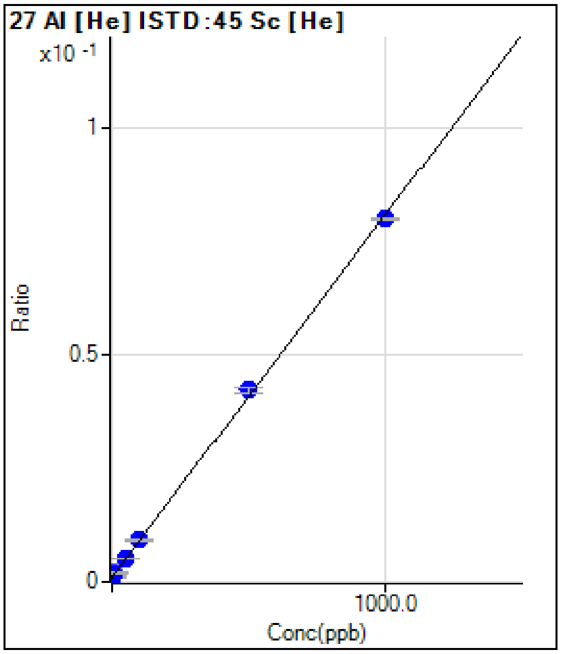

	Rjct	Conc.	Calc Conc.	CPS	Ratio	Det.	RSD
1	☐	0.000	0.000	197.79	0.0010	P	15.6
2	☑	0.500		307.79	0.0015	P	8.9
3	☑	1.000		344.46	0.0017	P	11.0
4	☑	2.500		376.69	0.0019	P	8.4
5	☑	5.000		407.80	0.0020	P	14.2
6	☐	10.000	11.058	386.69	0.0019	P	6.6
7	☐	50.000	50.809	1021.18	0.0050	P	4.0
8	☐	100.000	102.319	1844.61	0.0091	P	4.8
9	☐	500.000	518.237	8095.43	0.0422	P	3.0
10	☐	1000.000	990.598	14769.70	0.0798	P	0.9

y = 7.9584E-005 * x + 0.0010
R = 0.9998
DL = 5.905
BEC = 12.63

Weight: <None>
Min Conc: 0

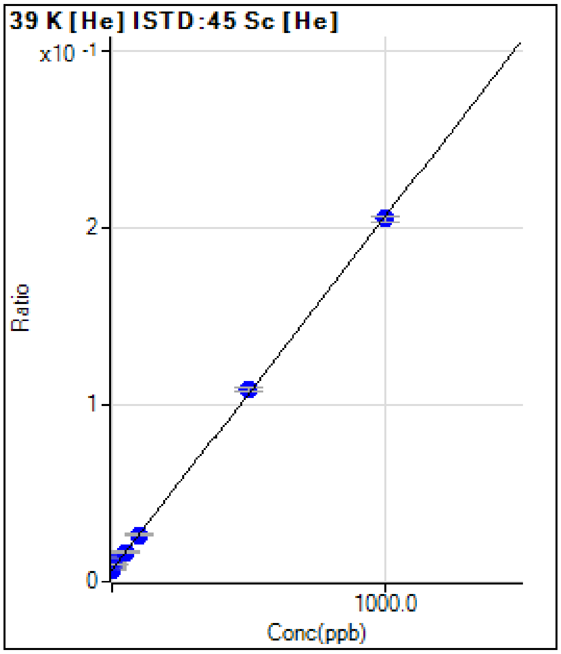

	Rjct	Conc.	Calc Conc.	CPS	Ratio	Det.	RSD
1	☐	0.000	0.000	1316.79	0.0067	P	9.8
2	☑	0.500		1621.26	0.0081	P	10.1
3	☑	1.000		2055.76	0.0103	P	5.7
4	☑	2.500		2605.86	0.0132	P	5.8
5	☑	5.000		2753.66	0.0136	P	3.9
6	☑	10.000		2009.10	0.0098	P	5.5
7	☐	50.000	48.728	3317.11	0.0164	P	6.4
8	☐	100.000	98.687	5315.42	0.0264	P	5.5
9	☐	500.000	511.430	20811.35	0.1086	P	2.0
10	☐	1000.000	994.480	37895.26	0.2049	P	1.3

y = 1.9926E-004 * x + 0.0067
R = 0.9999
DL = 9.902
BEC = 33.59

Weight: <None>
Min Conc: 0

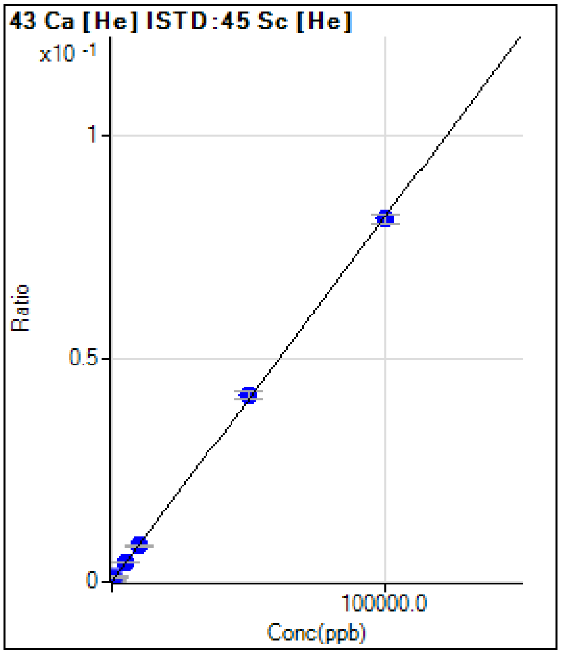

	Rjct	Conc.	Calc Conc.	CPS	Ratio	Det.	RSD
1	☐	0.000	0.000	12.22	0.0001	P	103.6
2	☑	50.000		53.33	0.0003	P	21.2
3	☑	100.000		61.12	0.0003	P	11.1
4	☑	250.000		138.90	0.0007	P	8.5
5	☑	500.000		143.34	0.0007	P	10.0
6	☑	1000.000		224.46	0.0011	P	29.5
7	☐	5000.000	5137.010	862.28	0.0043	P	4.0
8	☐	10000.000	9802.333	1629.03	0.0081	P	3.9
9	☐	50000.000	51117.349	8023.19	0.0419	P	3.9
10	☐	100000.000	99454.242	15056.70	0.0814	P	3.0

y = 8.1790E-007 * x + 6.2310E-005
R = 0.9999
DL = 236.7
BEC = 76.18

Weight: <None>
Min Conc: 0

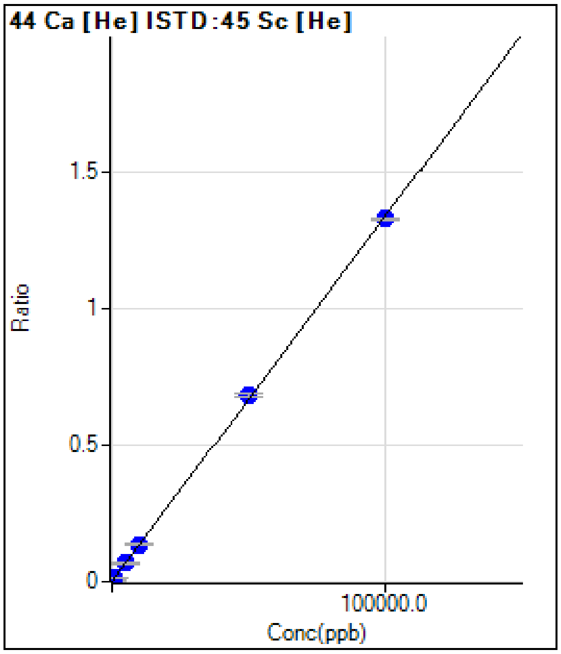

	Rjct	Conc.	Calc Conc.	CPS	Ratio	Det.	RSD
1	☐	0.000	0.000	167.79	0.0009	P	23.1
2	☑	50.000		1098.97	0.0055	P	0.2
3	☑	100.000		968.96	0.0049	P	10.8
4	☑	250.000		1947.97	0.0099	P	8.0
5	☑	500.000		2423.60	0.0119	P	1.5
6	☐	1000.000	999.706	2919.25	0.0142	P	3.2
7	☐	5000.000	5013.795	13744.54	0.0680	P	2.7
8	☐	10000.000	10215.271	27745.06	0.1376	P	1.5
9	☐	50000.000	51128.198	131313.29	0.6853	P	2.2
10	☐	100000.000	99413.687	246341.54	1.3316	P	0.8

y = 1.3386E-005 * x + 8.5300E-004
R = 0.9999
DL = 44.2
BEC = 63.72

Weight: <None>
Min Conc: 0

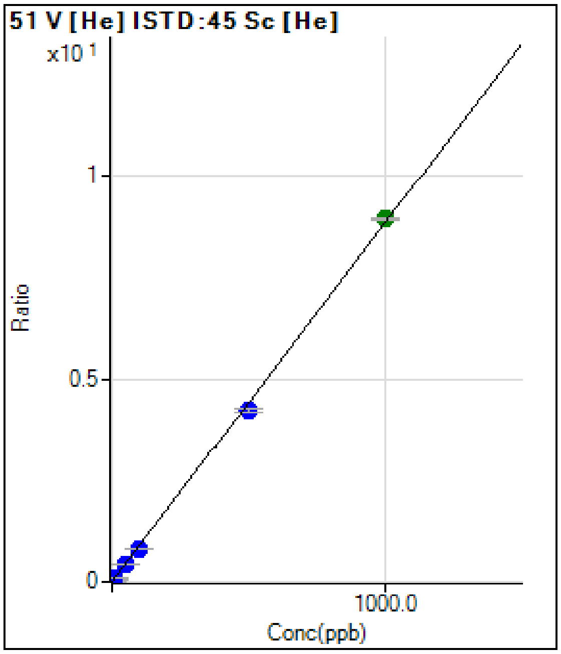

	Rjct	Conc.	Calc Conc.	CPS	Ratio	Det.	RSD
1	☐	0.000	0.000	113.34	0.0006	P	12.9
2	☐	0.500	0.697	1351.22	0.0068	P	0.5
3	☐	1.000	1.254	2322.47	0.0117	P	0.3
4	☐	2.500	2.528	4514.08	0.0230	P	5.4
5	☐	5.000	4.681	8526.81	0.0420	P	2.6
6	☐	10.000	8.318	15222.48	0.0742	P	0.7
7	☐	50.000	45.788	82104.83	0.4060	P	0.7
8	☐	100.000	92.270	164854.97	0.8176	P	1.2
9	☐	500.000	478.276	811619.19	4.2354	P	2.0
10	☐	1000.000	1011.863	1657616.42	8.9599	A	0.3

y = 0.0089 * x + 5.7617E-004
R = 0.9997
DL = 0.02525
BEC = 0.06507

Weight: <None>
Min Conc: 0

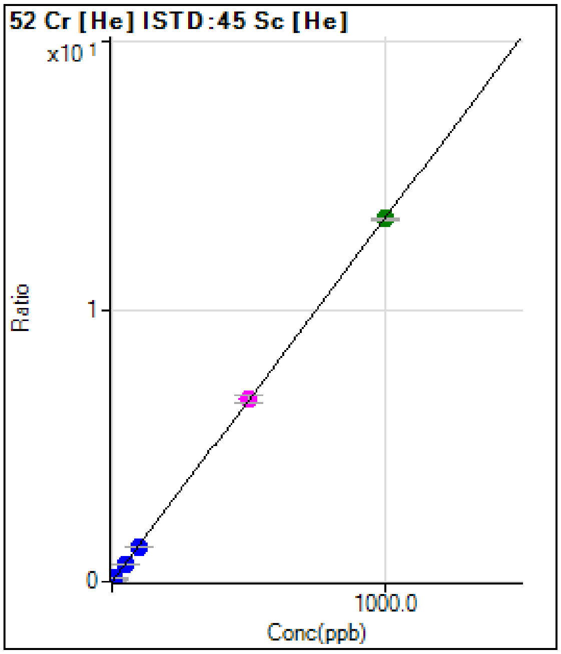

	Rjct	Conc.	Calc Conc.	CPS	Ratio	Det.	RSD
1	☐	0.000	0.000	505.40	0.0026	P	4.0
2	☑	0.500		3105.69	0.0155	P	0.7
3	☐	1.000	1.175	3653.27	0.0184	P	2.6
4	☐	2.500	3.689	10244.18	0.0522	P	9.2
5	☐	5.000	4.864	13786.77	0.0679	P	1.2
6	☐	10.000	8.353	23553.23	0.1148	P	0.3
7	☐	50.000	46.707	127484.22	0.6304	P	0.6
8	☐	100.000	94.839	257572.64	1.2774	P	1.1
9	☐	500.000	503.871	1298334.18	6.7755	M	4.0
10	☐	1000.000	998.759	2484084.37	13.4276	A	0.2

y = 0.0134 * x + 0.0026
R = 1.0000
DL = 0.02309
BEC = 0.1911

Weight: <None>
Min Conc: 0

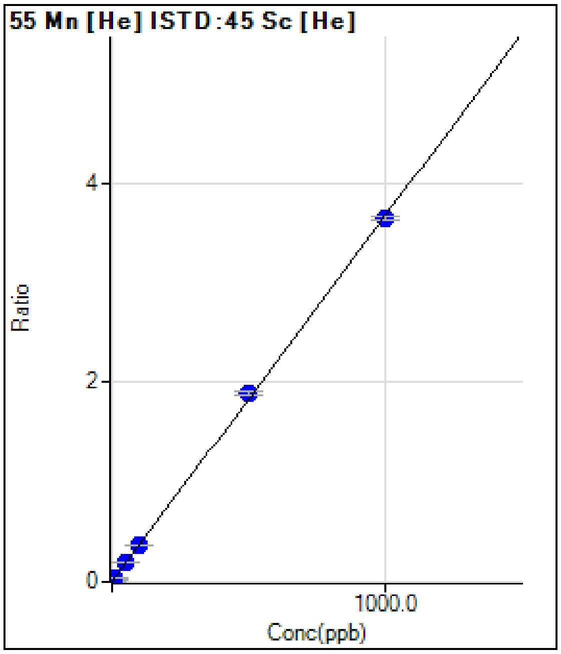

	Rjct	Conc.	Calc Conc.	CPS	Ratio	Det.	RSD
1	☐	0.000	0.000	175.56	0.0009	P	1.9
2	☑	0.500		1111.19	0.0056	P	3.9
3	☑	1.000		1299.00	0.0065	P	8.2
4	☑	2.500		8213.30	0.0418	P	10.4
5	☐	5.000	5.730	4444.06	0.0219	P	2.4
6	☐	10.000	9.125	7043.86	0.0343	P	2.5
7	☐	50.000	50.129	37346.91	0.1847	P	0.4
8	☐	100.000	100.959	74812.35	0.3710	P	1.3
9	☐	500.000	514.384	361520.41	1.8866	P	2.4
10	☐	1000.000	992.711	673378.05	3.6402	P	1.2

y = 0.0037 * x + 8.9236E-004
R = 0.9999
DL = 0.01364
BEC = 0.2434

Weight: <None>
Min Conc: 0

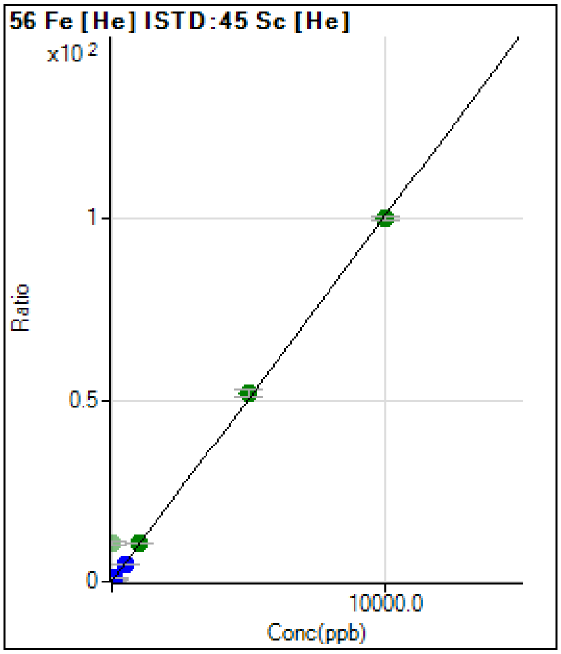

	Rjct	Conc.	Calc Conc.	CPS	Ratio	Det.	RSD
1	☐	0.000	0.000	29888.80	0.1519	P	0.7
2	☑	5.000		123190.42	0.6155	P	0.2
3	☑	10.000		80949.63	0.4070	P	0.7
4	☑	25.000		2041596.91	10.3963	A	10.4
5	☐	50.000	61.165	155678.47	0.7672	P	0.8
6	☐	100.000	88.833	214422.18	1.0455	P	0.5
7	☐	500.000	471.584	990062.32	4.8957	P	0.8
8	☐	1000.000	1029.455	2118759.28	10.5075	A	1.3
9	☐	5000.000	5148.895	9953307.26	51.9461	A	3.1
10	☐	10000.000	9924.084	18495681.59	99.9812	A	1.0

y = 0.0101 * x + 0.1519
R = 0.9998
DL = 0.3369
BEC = 15.1

Weight: <None>
Min Conc: 0

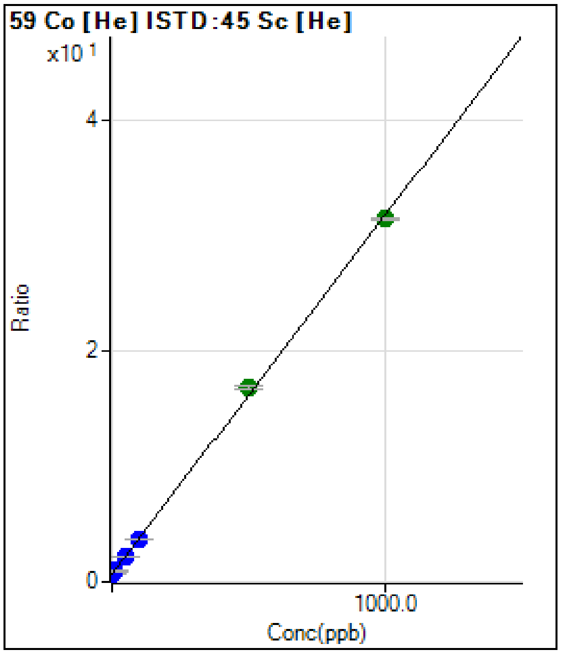

	Rjct	Conc.	Calc Conc.	CPS	Ratio	Det.	RSD
1	☐	0.000	0.000	127732.37	0.6493	P	0.7
2	☑	0.500		135910.83	0.6791	P	1.8
3	☑	1.000		140501.05	0.7064	P	1.1
4	☑	2.500		150613.16	0.7659	P	4.5
5	☐	5.000	5.448	166171.82	0.8189	P	0.6
6	☐	10.000	8.681	188583.30	0.9196	P	1.3
7	☐	50.000	47.783	432162.32	2.1370	P	1.5
8	☐	100.000	96.290	735466.25	3.6473	P	0.8
9	☐	500.000	519.746	3225468.87	16.8319	A	1.9
10	☐	1000.000	990.620	5826226.03	31.4929	A	0.7

y = 0.0311 * x + 0.6493
R = 0.9997
DL = 0.4234
BEC = 20.85

Weight: <None>
Min Conc: 0

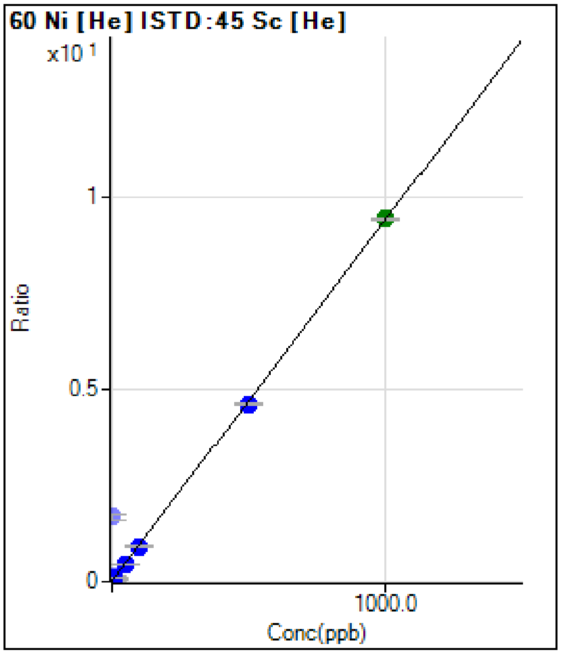

	Rjct	Conc.	Calc Conc.	CPS	Ratio	Det.	RSD
1	☐	0.000	0.000	3265.98	0.0166	P	3.5
2	☑	0.500		25458.62	0.1272	P	2.8
3	☑	1.000		8172.15	0.0411	P	1.4
4	☑	2.500		329206.34	1.6747	P	6.1
5	☐	5.000	4.504	11926.64	0.0588	P	0.9
6	☐	10.000	8.557	19838.18	0.0967	P	0.5
7	☐	50.000	46.888	92150.73	0.4557	P	0.8
8	☐	100.000	96.719	185976.64	0.9223	P	0.8
9	☐	500.000	491.586	885298.02	4.6199	P	2.1
10	☐	1000.000	1004.708	1743536.75	9.4249	A	0.7

y = 0.0094 * x + 0.0166
R = 1.0000
DL = 0.1875
BEC = 1.773

Weight: <None>
Min Conc: 0

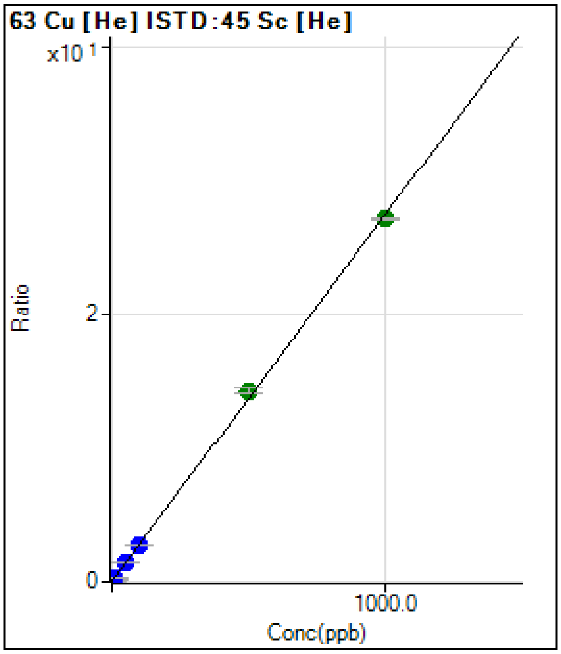

	Rjct	Conc.	Calc Conc.	CPS	Ratio	Det.	RSD
1	☐	0.000	0.000	4384.05	0.0223	P	1.4
2	☑	0.500		39967.77	0.1997	P	4.4
3	☑	1.000		17620.27	0.0886	P	1.4
4	☑	2.500		29757.45	0.1514	P	6.9
5	☐	5.000	5.773	36644.57	0.1806	P	0.6
6	☐	10.000	9.822	59802.44	0.2916	P	1.8
7	☐	50.000	49.427	278575.67	1.3775	P	1.2
8	☐	100.000	99.660	555507.50	2.7549	P	0.9
9	☐	500.000	521.177	2742559.02	14.3126	A	2.5
10	☐	1000.000	989.472	5023126.17	27.1528	A	0.4

y = 0.0274 * x + 0.0223
R = 0.9997
DL = 0.03454
BEC = 0.8127

Weight: <None>
Min Conc: 0

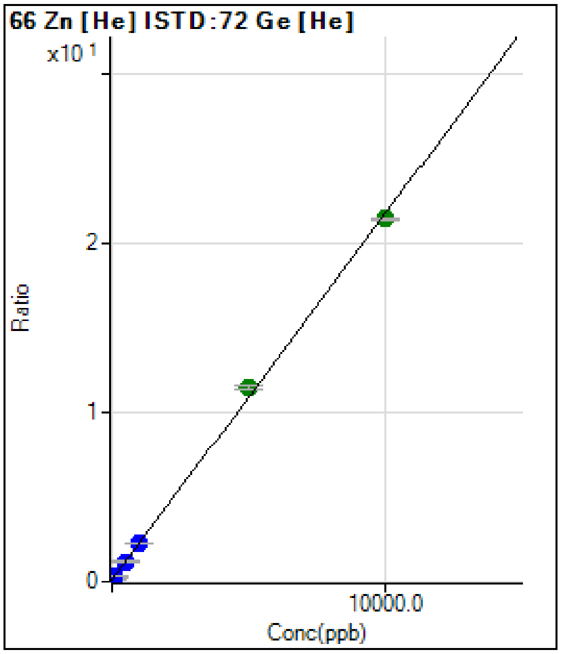

	Rjct	Conc.	Calc Conc.	CPS	Ratio	Det.	RSD
1	☐	0.000	0.000	20107.39	0.1015	P	3.2
2	☑	5.000		48065.77	0.2405	P	2.1
3	☑	10.000		32404.36	0.1634	P	0.7
4	☑	25.000		35858.62	0.1796	P	5.1
5	☐	50.000	57.105	45784.34	0.2249	P	0.7
6	☐	100.000	92.025	61160.46	0.3004	P	1.6
7	☐	500.000	503.481	238735.13	1.1895	P	1.8
8	☐	1000.000	1000.758	457565.15	2.2640	P	0.3
9	☐	5000.000	5264.688	2184700.49	11.4779	A	1.6
10	☐	10000.000	9867.450	3901598.17	21.4239	A	0.4

y = 0.0022 * x + 0.1015
R = 0.9995
DL = 4.562
BEC = 46.98

Weight: <None>
Min Conc: 0

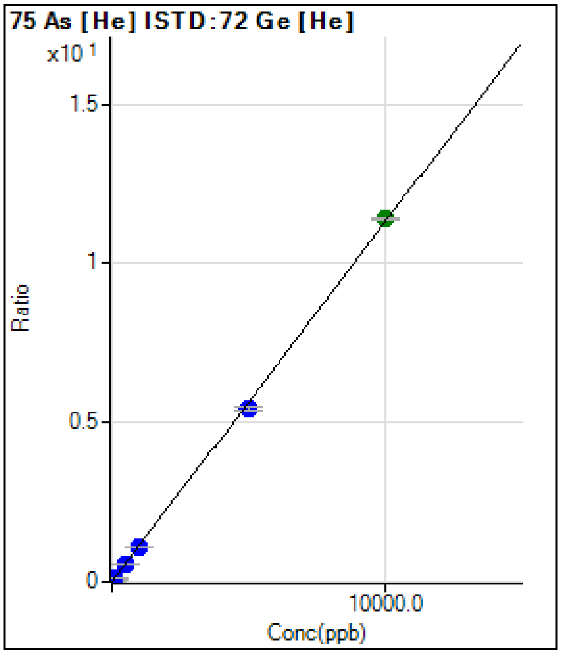

	Rjct	Conc.	Calc Conc.	CPS	Ratio	Det.	RSD
1	☐	0.000	0.000	21.88	0.0001	P	18.3
2	☐	5.000	7.494	1712.90	0.0086	P	3.0
3	☐	10.000	11.756	2654.44	0.0134	P	0.7
4	☐	25.000	23.979	5426.13	0.0272	P	6.5
5	☐	50.000	46.662	10745.38	0.0528	P	0.5
6	☐	100.000	83.888	19305.05	0.0948	P	0.9
7	☐	500.000	468.741	106230.39	0.5293	P	1.2
8	☐	1000.000	941.742	214884.38	1.0633	P	0.1
9	☐	5000.000	4826.612	1037147.78	5.4489	P	1.8
10	☐	10000.000	10094.260	2075293.02	11.3957	A	0.3

y = 0.0011 * x + 1.1044E-004
R = 0.9998
DL = 0.05377
BEC = 0.09783

Weight: <None>
Min Conc: 0

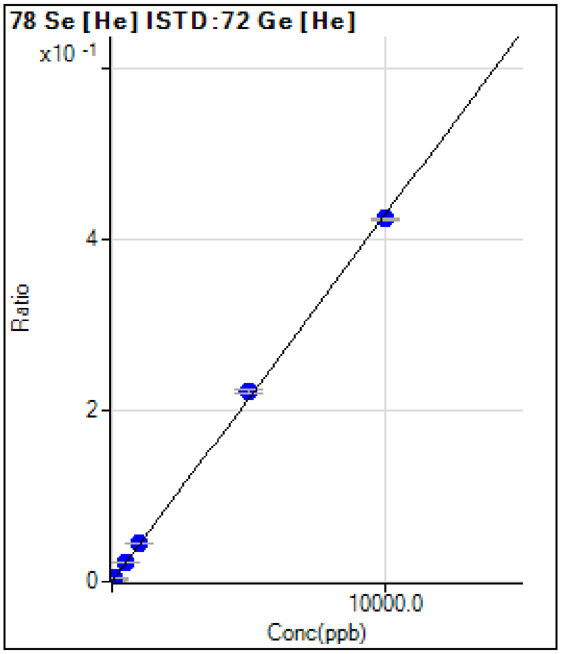

	Rjct	Conc.	Calc Conc.	CPS	Ratio	Det.	RSD
1	☐	0.000	0.000	0.87	0.0000	P	26.9
2	☑	5.000		67.86	0.0003	P	10.6
3	☑	10.000		120.56	0.0006	P	2.6
4	☐	25.000	26.054	224.02	0.0011	P	4.3
5	☐	50.000	52.116	455.70	0.0022	P	2.0
6	☐	100.000	92.338	806.94	0.0040	P	1.3
7	☐	500.000	515.896	4440.08	0.0221	P	1.0
8	☐	1000.000	1032.824	8949.66	0.0443	P	1.1
9	☐	5000.000	5200.475	42437.90	0.2230	P	1.8
10	☐	10000.000	9895.748	77261.79	0.4243	P	0.7

y = 4.2872E-005 * x + 4.3779E-006
R = 0.9997
DL = 0.08234
BEC = 0.1021

Weight: <None>
Min Conc: 0

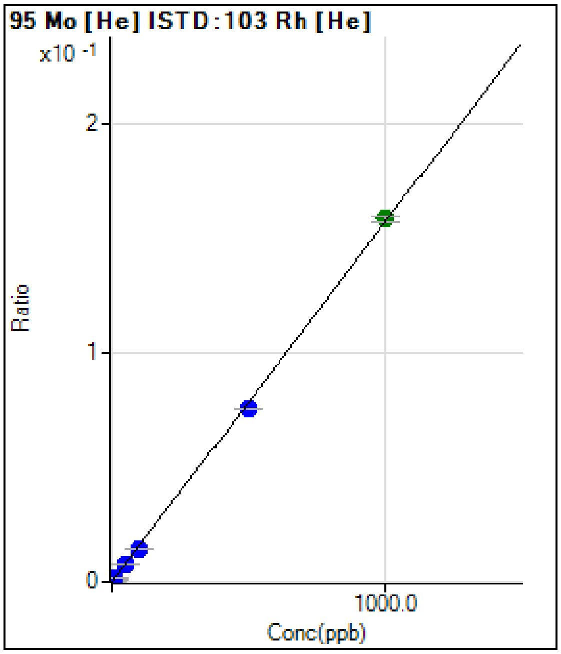

	Rjct	Conc.	Calc Conc.	CPS	Ratio	Det.	RSD
1	☐	0.000	0.000	57.78	0.0000	P	6.9
2	☐	0.500	0.737	1801.29	0.0001	P	1.8
3	☐	1.000	1.168	2835.90	0.0002	P	6.9
4	☐	2.500	2.516	6015.73	0.0004	P	5.7
5	☐	5.000	4.682	11446.34	0.0007	P	0.9
6	☐	10.000	8.092	19835.20	0.0013	P	2.0
7	☐	50.000	46.067	111177.18	0.0072	P	0.5
8	☐	100.000	92.507	224753.03	0.0145	P	1.1
9	☐	500.000	482.513	1099309.79	0.0756	P	0.7
10	☐	1000.000	1009.710	2209959.81	0.1583	A	1.4

y = 1.5672E-004 * x + 3.8866E-006
R = 0.9998
DL = 0.005124
BEC = 0.0248

Weight: <None>
Min Conc: 0

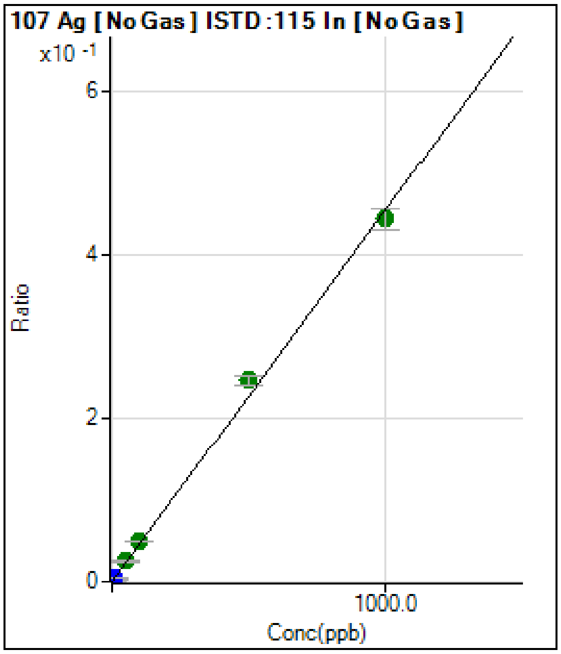

	Rjct	Conc.	Calc Conc.	CPS	Ratio	Det.	RSD
1	☐	0.000	0.000	2068.00	0.0000	P	5.7
2	☑	0.500		39988.95	0.0005	P	1.8
3	☑	1.000		64477.20	0.0007	P	0.3
4	☐	2.500	2.608	109068.05	0.0012	P	4.6
5	☐	5.000	5.605	230451.37	0.0026	P	0.9
6	☐	10.000	9.399	382761.93	0.0043	P	0.2
7	☐	50.000	54.636	2211566.25	0.0248	A	1.3
8	☐	100.000	106.991	4367369.21	0.0486	A	0.5
9	☐	500.000	542.195	21249706.08	0.2463	A	5.5
10	☐	1000.000	977.974	38508481.93	0.4443	A	5.8

y = 4.5428E-004 * x + 2.4769E-005
R = 0.9988
DL = 0.009281
BEC = 0.05452

Weight: <None>
Min Conc: 0

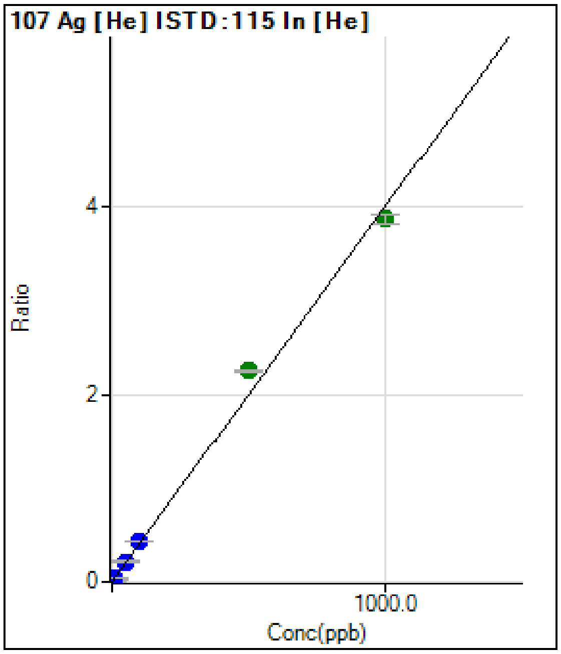

	Rjct	Conc.	Calc Conc.	CPS	Ratio	Det.	RSD
1	☐	0.000	0.000	355.57	0.0002	P	5.5
2	☑	0.500		7858.74	0.0040	P	1.6
3	☑	1.000		13056.51	0.0067	P	0.8
4	☐	2.500	2.832	22288.43	0.0115	P	4.5
5	☐	5.000	5.754	45865.40	0.0232	P	0.5
6	☐	10.000	9.618	76536.61	0.0387	P	0.7
7	☐	50.000	53.647	421495.98	0.2150	P	1.1
8	☐	100.000	108.477	849568.68	0.4345	P	0.9
9	☐	500.000	562.053	4153357.20	2.2503	A	0.7
10	☐	1000.000	967.943	6925527.40	3.8753	A	2.6

y = 0.0040 * x + 1.8233E-004
R = 0.9974
DL = 0.007531
BEC = 0.04554

Weight: <None>
Min Conc: 0

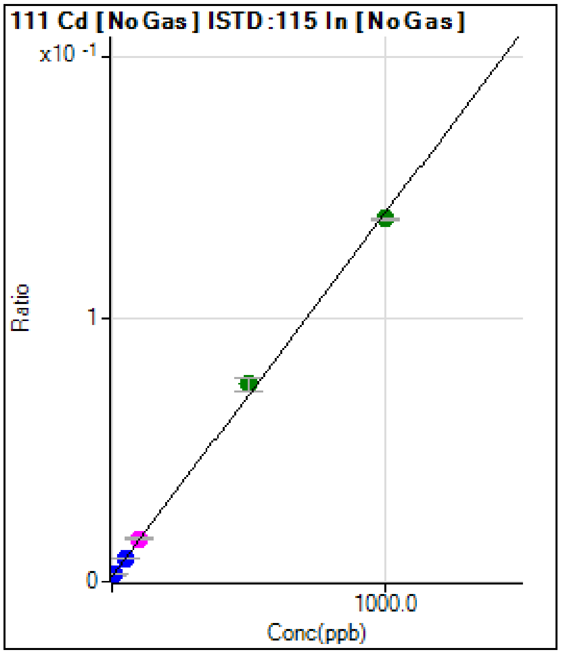

	Rjct	Conc.	Calc Conc.	CPS	Ratio	Det.	RSD
1	☐	0.000	0.000	166910.17	0.0020	P	0.8
2	☑	0.500		184869.20	0.0021	P	1.0
3	☑	1.000		193669.59	0.0022	P	1.8
4	☐	2.500	2.424	210580.78	0.0023	P	3.4
5	☐	5.000	5.203	243636.19	0.0027	P	0.8
6	☐	10.000	8.901	287775.47	0.0032	P	1.1
7	☐	50.000	49.136	782022.51	0.0088	P	0.3
8	☐	100.000	103.269	1460473.62	0.0163	M	3.0
9	☐	500.000	529.117	6474763.86	0.0751	A	6.2
10	☐	1000.000	985.168	11965366.49	0.1381	A	0.6

y = 1.3810E-004 * x + 0.0020
R = 0.9994
DL = 0.3623
BEC = 14.48

Weight: <None>
Min Conc: 0

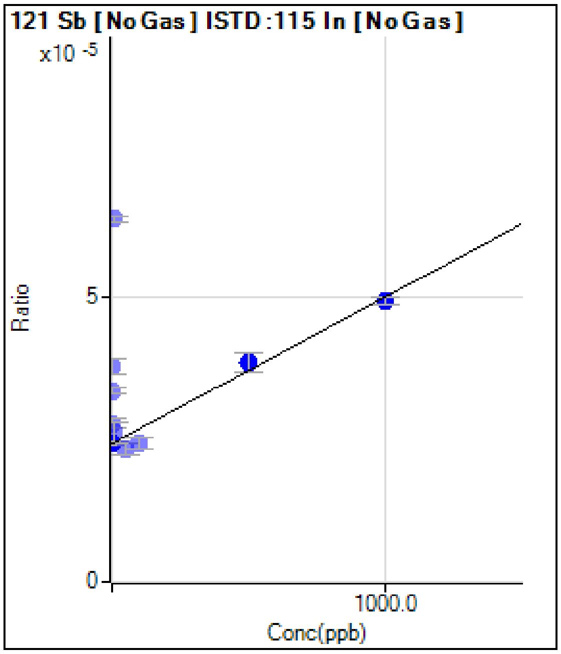

	Rjct	Conc.	Calc Conc.	CPS	Ratio	Det.	RSD
1	☐	0.000	0.000	2031.33	0.0000	P	3.8
2	☑	0.500		2892.59	0.0000	P	2.9
3	☑	1.000		2422.60	0.0000	P	6.1
4	☑	2.500		3428.29	0.0000	P	7.0
5	☑	5.000		5725.65	0.0001	P	1.5
6	☑	10.000		2362.52	0.0000	P	13.0
7	☑	50.000		2091.33	0.0000	P	8.8
8	☑	100.000		2188.02	0.0000	P	9.0
9	☐	500.000	551.717	3323.81	0.0000	P	9.0
10	☐	1000.000	974.142	4287.39	0.0000	P	2.5

y = 2.5798E-008 * x + 2.4334E-005
R = 0.9971
DL = 107.7
BEC = 943.3

Weight: <None>
Min Conc: 0

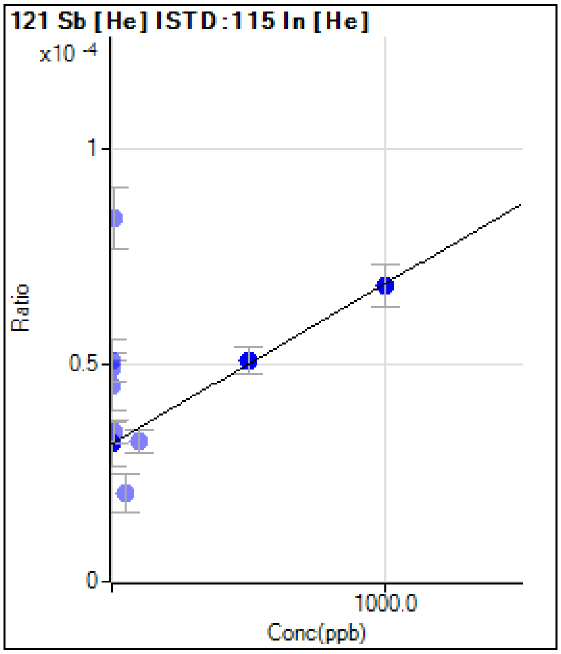

	Rjct	Conc.	Calc Conc.	CPS	Ratio	Det.	RSD
1	☐	0.000	0.000	62.23	0.0000	P	32.0
2	☑	0.500		97.78	0.0000	P	13.8
3	☑	1.000		87.78	0.0000	P	25.8
4	☑	2.500		98.89	0.0001	P	19.5
5	☑	5.000		165.57	0.0001	P	16.8
6	☑	10.000		68.89	0.0000	P	15.3
7	☑	50.000		40.00	0.0000	P	44.1
8	☑	100.000		63.34	0.0000	P	15.5
9	☐	500.000	521.074	94.45	0.0001	P	11.6
10	☐	1000.000	989.463	122.23	0.0001	P	14.6

y = 3.6972E-008 * x + 3.1878E-005
R = 0.9995
DL = 828.2
BEC = 862.2

Weight: <None>
Min Conc: 0

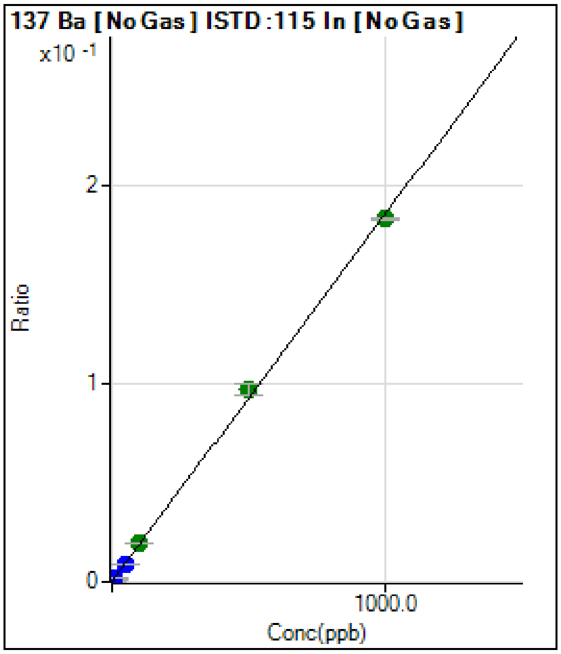

	Rjct	Conc.	Calc Conc.	CPS	Ratio	Det.	RSD
1	☐	0.000	0.000	10996.37	0.0001	P	1.6
2	☑	0.500		32416.24	0.0004	P	1.9
3	☑	1.000		39670.27	0.0005	P	1.7
4	☑	2.500		65834.33	0.0007	P	3.5
5	☐	5.000	5.552	104186.97	0.0012	P	1.5
6	☐	10.000	9.190	163792.03	0.0018	P	1.2
7	☐	50.000	48.347	810592.34	0.0091	P	0.7
8	☐	100.000	102.710	1724135.67	0.0192	A	0.5
9	☐	500.000	523.683	8396460.85	0.0973	A	5.8
10	☐	1000.000	987.976	15907071.43	0.1835	A	0.8

y = 1.8563E-004 * x + 1.3172E-004
R = 0.9996
DL = 0.03303
BEC = 0.7096

Weight: <None>
Min Conc: 0

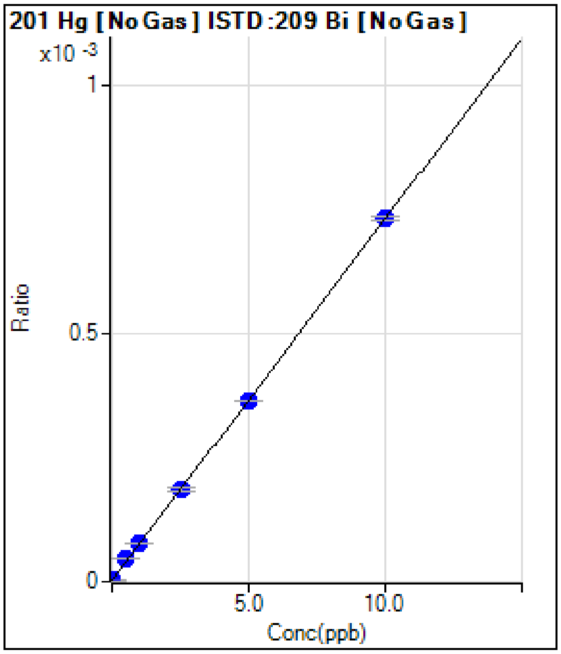

	Rjct	Conc.	Calc Conc.	CPS	Ratio	Det.	RSD
1	☐	0.000	0.000	178.45	0.0000	P	8.2
2	☐	0.500	0.591	3161.96	0.0000	P	1.5
3	☐	1.000	1.015	5370.31	0.0001	P	1.3
4	☐	2.500	2.530	13423.48	0.0002	P	4.0
5	☐	5.000	4.950	26182.39	0.0004	P	0.0
6	☐	10.000	10.011	52513.90	0.0007	P	1.0
7	☑	0.000		229.64	0.0000	P	7.8
8	☑	0.000		163.30	0.0000	P	7.0
9	☑	0.000		145.46	0.0000	P	7.7
10	☑	0.000		145.46	0.0000	P	8.4

y = 7.2748E-005 * x + 2.7199E-006
R = 0.9999
DL = 0.009159
BEC = 0.03739

Weight: <None>
Min Conc: 0

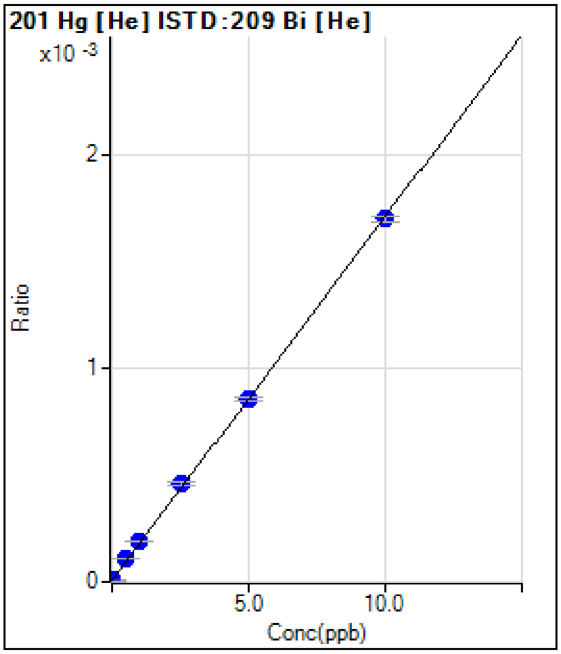

	Rjct	Conc.	Calc Conc.	CPS	Ratio	Det.	RSD
1	☐	0.000	0.000	56.57	0.0000	P	30.7
2	☐	0.500	0.593	1043.48	0.0001	P	4.3
3	☐	1.000	1.057	1800.12	0.0002	P	1.3
4	☐	2.500	2.666	4461.28	0.0005	P	4.7
5	☐	5.000	5.004	8473.68	0.0009	P	2.3
6	☐	10.000	9.946	16918.34	0.0017	P	1.3
7	☑	0.000		66.33	0.0000	P	8.9
8	☑	0.000		47.81	0.0000	P	19.1
9	☑	0.000		42.09	0.0000	P	20.7
10	☑	0.000		35.01	0.0000	P	10.2

y = 1.7012E-004 * x + 5.9554E-006
R = 0.9999
DL = 0.03225
BEC = 0.03501

Weight: <None>
Min Conc: 0

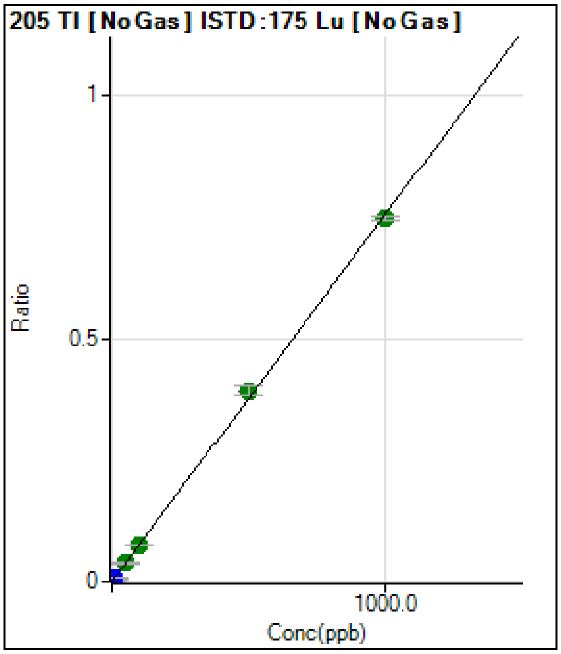

	Rjct	Conc.	Calc Conc.	CPS	Ratio	Det.	RSD
1	☐	0.000	0.000	705.60	0.0000	P	12.0
2	☑	0.500		74084.09	0.0006	P	1.1
3	☑	1.000		127212.13	0.0010	P	0.9
4	☐	2.500	2.378	243041.50	0.0018	P	3.5
5	☐	5.000	4.939	502960.73	0.0037	P	1.1
6	☐	10.000	8.855	890687.22	0.0067	P	0.2
7	☐	50.000	49.981	5029088.88	0.0377	A	1.2
8	☐	100.000	100.052	10136302.56	0.0754	A	0.7
9	☐	500.000	521.536	50372850.36	0.3932	A	4.9
10	☐	1000.000	989.240	96208338.56	0.7459	A	1.2

y = 7.5396E-004 * x + 5.6445E-006
R = 0.9997
DL = 0.002689
BEC = 0.007486

Weight: <None>
Min Conc: 0

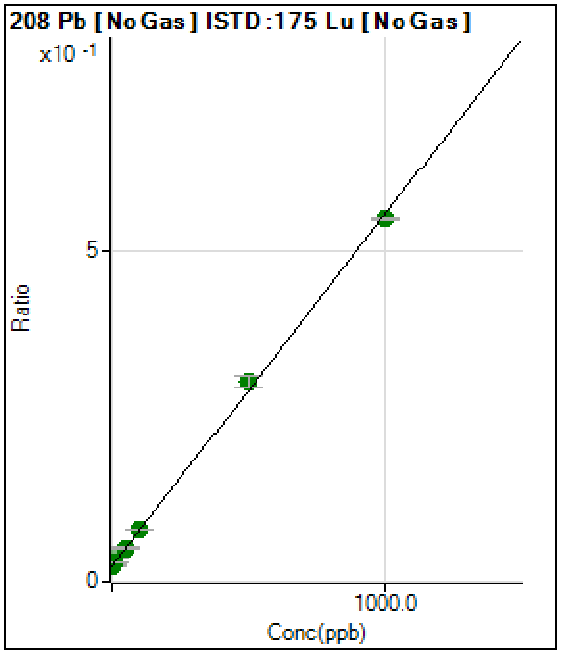

	Rjct	Conc.	Calc Conc.	CPS	Ratio	Det.	RSD
1	☐	0.000	0.000	2982326.90	0.0238	A	0.8
2	☑	0.500		3362735.02	0.0261	A	0.0
3	☑	1.000		3450507.72	0.0264	A	0.4
4	☑	2.500		3534733.77	0.0262	A	3.6
5	☑	5.000		4633405.35	0.0344	A	0.8
6	☐	10.000	10.637	3931830.88	0.0295	A	0.4
7	☐	50.000	50.568	6769028.51	0.0507	A	1.1
8	☐	100.000	100.775	10402566.02	0.0774	A	0.5
9	☐	500.000	525.597	38841648.03	0.3033	A	6.0
10	☐	1000.000	987.089	70776107.83	0.5487	A	0.5

y = 5.3170E-004 * x + 0.0238
R = 0.9995
DL = 1.068
BEC = 44.84

Weight: <None>
Min Conc: 0

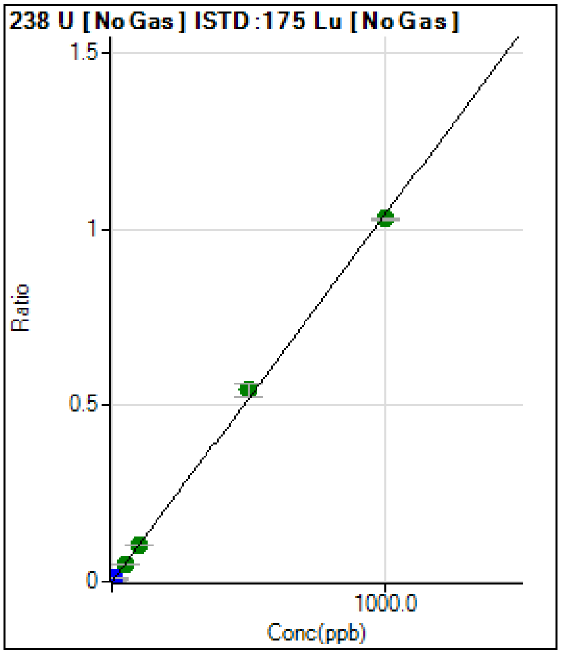

	Rjct	Conc.	Calc Conc.	CPS	Ratio	Det.	RSD
1	☐	0.000	0.000	401.13	0.0000	P	11.3
2	☐	0.500	0.741	99865.85	0.0008	P	0.6
3	☐	1.000	1.239	169255.58	0.0013	P	0.8
4	☐	2.500	2.310	325439.07	0.0024	P	4.2
5	☐	5.000	4.911	689912.20	0.0051	P	0.8
6	☐	10.000	8.577	1190705.86	0.0089	P	0.5
7	☐	50.000	49.292	6848837.40	0.0513	A	0.5
8	☐	100.000	98.640	13799274.38	0.1027	A	0.3
9	☐	500.000	522.286	69625962.30	0.5438	A	6.7
10	☐	1000.000	989.043	132832193.58	1.0298	A	0.8

y = 0.0010 * x + 3.2086E-006
R = 0.9997
DL = 0.001041
BEC = 0.003082

Weight: <None>
Min Conc: 0

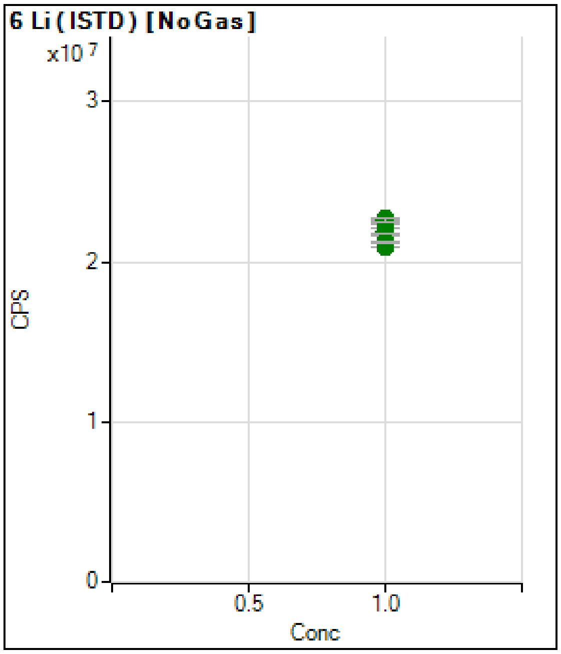

	Rjct	Conc.	Calc Conc.	CPS	Ratio	Det.	RSD
1	☐	1.000		20911891.44		A	0.5
2	☐	1.000		21198230.72		A	0.8
3	☐	1.000		21661787.70		A	0.7
4	☐	1.000		21859816.16		A	1.7
5	☐	1.000		22503963.34		A	1.1
6	☐	1.000		22666683.58		A	0.4
7	☐	1.000		22340204.76		A	0.3
8	☐	1.000		22565101.42		A	0.7
9	☐	1.000		21301305.74		A	3.6
10	☐	1.000		21196115.64		A	0.3

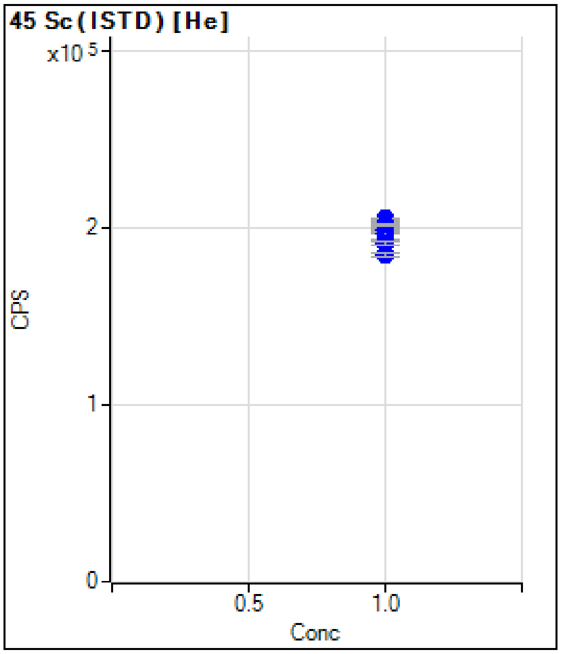

	Rjct	Conc.	Calc Conc.	CPS	Ratio	Det.	RSD
1	☐	1.000		196732.44		P	0.4
2	☐	1.000		200154.52		P	0.9
3	☐	1.000		198912.25		P	0.7
4	☐	1.000		196876.23		P	3.7
5	☐	1.000		202916.02		P	0.1
6	☐	1.000		205085.77		P	0.4
7	☐	1.000		202234.86		P	0.5
8	☐	1.000		201651.11		P	0.6
9	☐	1.000		191660.79		P	1.3
10	☐	1.000		185001.73		P	1.4

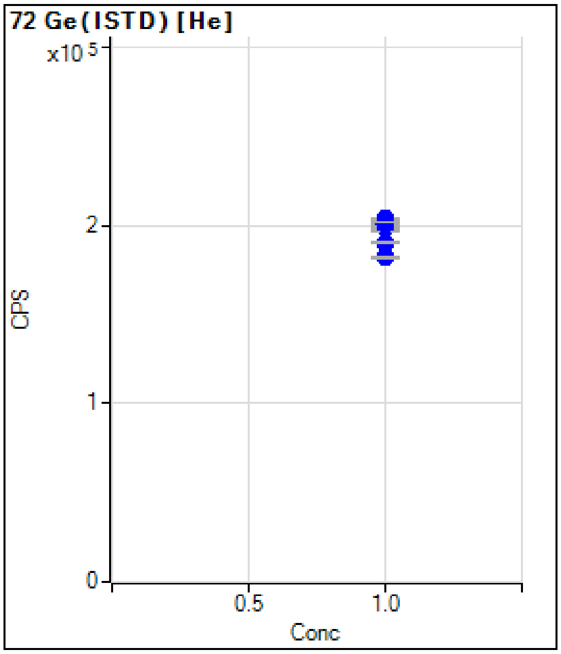

	Rjct	Conc.	Calc Conc.	CPS	Ratio	Det.	RSD
1	☐	1.000		198068.05		P	0.3
2	☐	1.000		199850.03		P	0.8
3	☐	1.000		198361.33		P	0.2
4	☐	1.000		199921.03		P	3.3
5	☐	1.000		203560.98		P	0.4
6	☐	1.000		203613.85		P	0.2
7	☐	1.000		200724.02		P	1.0
8	☐	1.000		202100.35		P	0.1
9	☐	1.000		190357.99		P	0.9
10	☐	1.000		182112.35		P	0.4

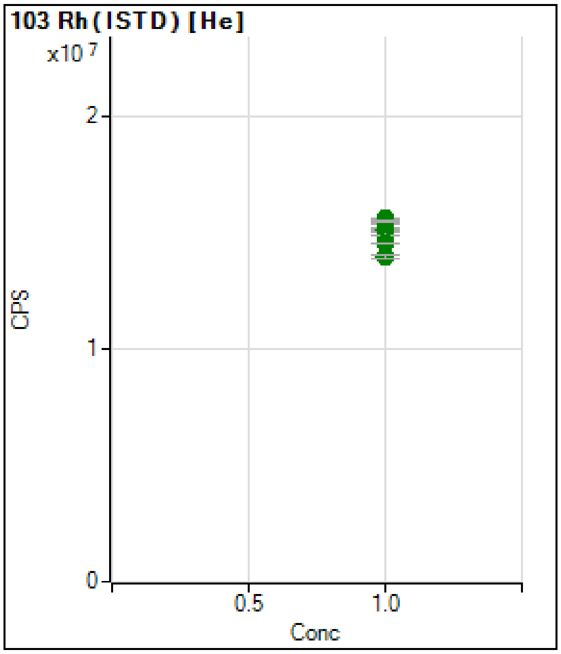

	Rjct	Conc.	Calc Conc.	CPS	Ratio	Det.	RSD
1	☐	1.000		14868123.11		A	0.4
2	☐	1.000		15093278.52		A	0.7
3	☐	1.000		15167967.97		A	0.4
4	☐	1.000		15122187.14		A	3.1
5	☐	1.000		15518670.60		A	0.9
6	☐	1.000		15592178.93		A	0.5
7	☐	1.000		15390411.72		A	0.2
8	☐	1.000		15498806.44		A	0.7
9	☐	1.000		14536079.09		A	0.2
10	☐	1.000		13967034.10		A	1.6

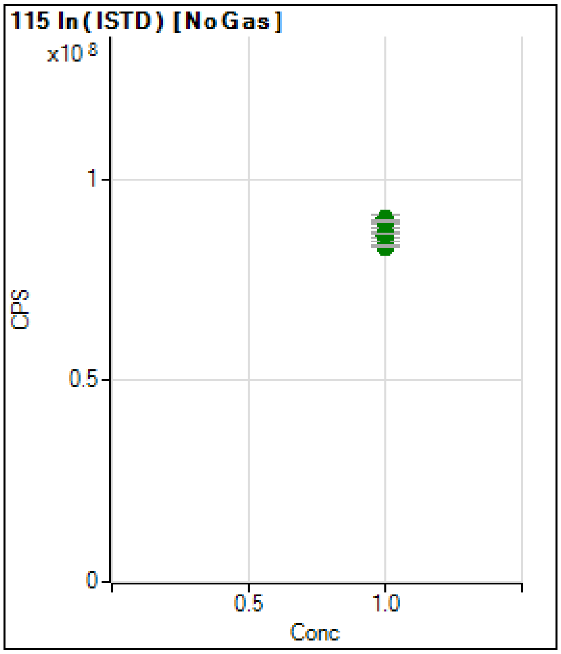

	Rjct	Conc.	Calc Conc.	CPS	Ratio	Det.	RSD
1	☐	1.000		83476407.65		A	0.7
2	☐	1.000		86142403.16		A	0.7
3	☐	1.000		86822387.60		A	0.8
4	☐	1.000		90258910.88		A	2.5
5	☐	1.000		89633220.89		A	0.3
6	☐	1.000		89132513.12		A	0.6
7	☐	1.000		89015289.79		A	0.6
8	☐	1.000		89808577.55		A	0.6
9	☐	1.000		86394284.82		A	4.1
10	☐	1.000		86672435.38		A	0.2

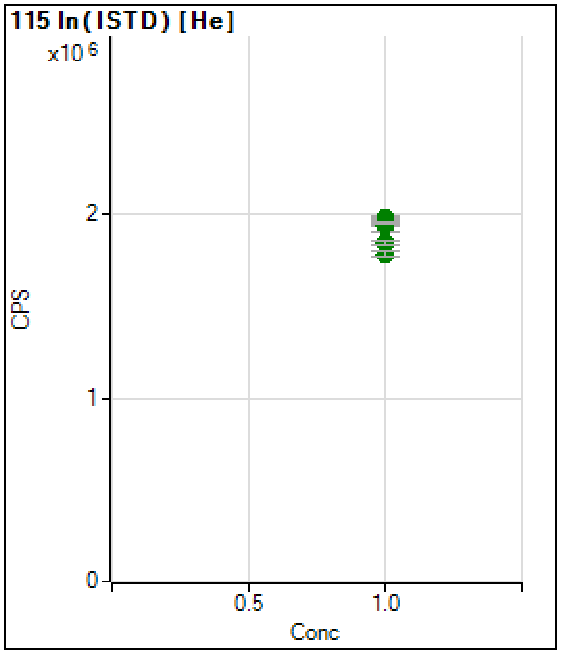

	Rjct	Conc.	Calc Conc.	CPS	Ratio	Det.	RSD
1	☐	1.000		1950427.00		A	0.8
2	☐	1.000		1974351.31		A	1.4
3	☐	1.000		1939916.78		A	0.5
4	☐	1.000		1936705.32		A	3.1
5	☐	1.000		1975439.80		A	0.5
6	☐	1.000		1978437.68		A	0.6
7	☐	1.000		1960918.50		A	0.3
8	☐	1.000		1955552.30		A	0.9
9	☐	1.000		1845600.86		A	1.0
10	☐	1.000		1787272.87		A	1.8

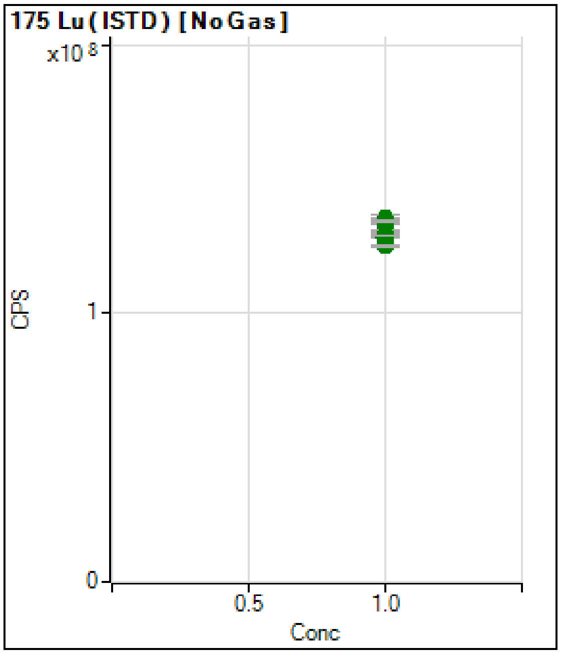

	Rjct	Conc.	Calc Conc.	CPS	Ratio	Det.	RSD
1	☐	1.000		125094654.80		A	1.0
2	☐	1.000		128982881.41		A	1.1
3	☐	1.000		130922204.72		A	0.4
4	☐	1.000		135224804.65		A	2.6
5	☐	1.000		134855651.32		A	0.7
6	☐	1.000		133293665.79		A	0.2
7	☐	1.000		133443754.68		A	0.7
8	☐	1.000		134360491.33		A	0.4
9	☐	1.000		128276094.76		A	4.2
10	☐	1.000		128994241.41		A	0.3

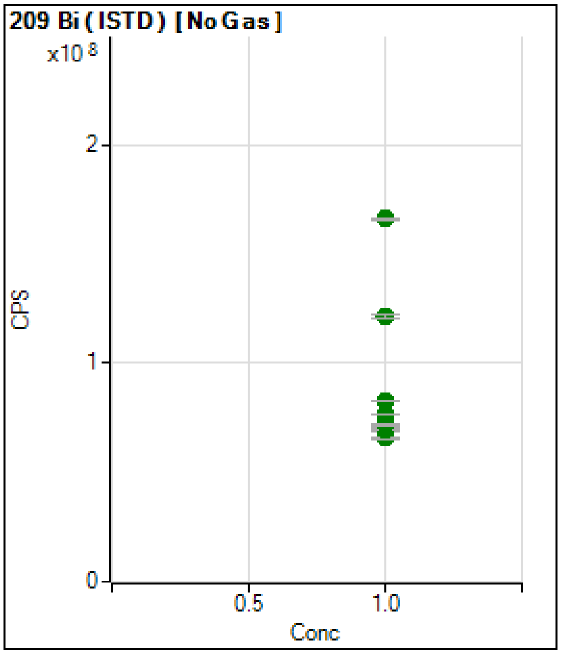

	Rjct	Conc.	Calc Conc.	CPS	Ratio	Det.	RSD
1	☐	1.000		65629935.69		A	0.5
2	☐	1.000		69137003.97		A	0.6
3	☐	1.000		70160344.51		A	0.5
4	☐	1.000		71894830.04		A	1.5
5	☐	1.000		72157788.92		A	0.4
6	☐	1.000		71836165.60		A	0.4
7	☐	1.000		76584509.42		A	0.4
8	☐	1.000		82531163.77		A	0.2
9	☐	1.000		121515424.86		A	1.0
10	☐	1.000		166142891.97		A	0.3

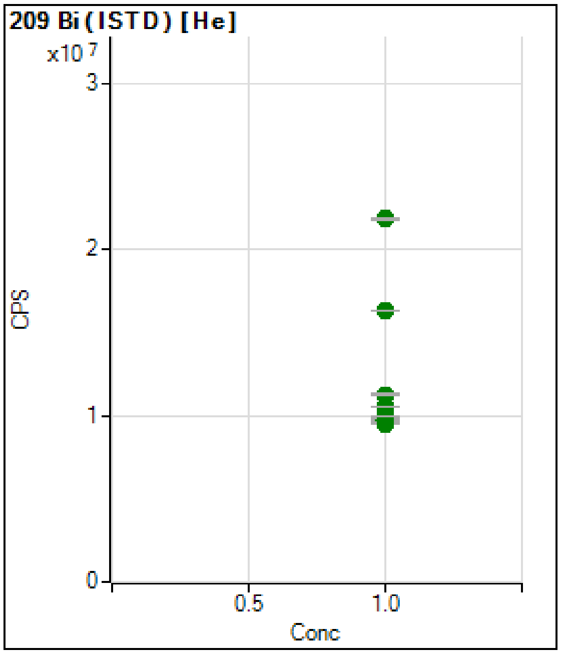

	Rjct	Conc.	Calc Conc.	CPS	Ratio	Det.	RSD
1	☐	1.000		9491104.72		A	0.4
2	☐	1.000		9772339.92		A	0.8
3	☐	1.000		9691408.12		A	0.1
4	☐	1.000		9717644.16		A	2.4
5	☐	1.000		9884069.44		A	0.6
6	☐	1.000		9963740.41		A	0.4
7	☐	1.000		10506100.12		A	0.4
8	☐	1.000		11280126.08		A	0.2
9	☐	1.000		16324264.76		A	0.6
10	☐	1.000		21839514.40		A	0.8
